# High efficiency and fast van der Waals hetero-photodiodes with a unilateral depletion region

**DOI:** 10.1038/s41467-019-12707-3

**Published:** 2019-10-11

**Authors:** Feng Wu, Qing Li, Peng Wang, Hui Xia, Zhen Wang, Yang Wang, Man Luo, Long Chen, Fansheng Chen, Jinshui Miao, Xiaoshuang Chen, Wei Lu, Chongxin Shan, Anlian Pan, Xing Wu, Wencai Ren, Deep Jariwala, Weida Hu

**Affiliations:** 1 State Key Laboratory of Infrared Physics, Key Laboratory of Intelligent Infrared Perception, Shanghai Institute of Technical Physics, Chinese Academy of Sciences, 200083 Shanghai, China; 2 University of Chinese Academy of Sciences, Chinese Academy of Sciences, 100049 Beijing, China; 3 Shenyang National Laboratory for Materials Science, Institute of Metal Research, Chinese Academy of Sciences, 110016 Shenyang, China; 4 Department of Electrical and Systems Engineering, University of Pennsylvania, Philadelphia, PA 19104 USA; 5 Henan Key Laboratory of Diamond Optoelectronic Materials and Devices, School of Physics and Engineering, Zhengzhou University, 45000 Zhengzhou, China; 6 Key Laboratory for Micro-Nano Physics and Technology of Hunan Province, and School of Physics and Electronics, Hunan University, 410082 Changsha, China; 7 School of Information Science and Technology, East China Normal University, 200083 Shanghai, China; 8 Hangzhou Institute for Advanced Study, University of Chinese Academy of Sciences, 310024 Hangzhou, China

**Keywords:** Materials for devices, Materials for devices, Two-dimensional materials, Two-dimensional materials, Electronics, photonics and device physics

## Abstract

Van der Waals (vdW) heterodiodes based on two-dimensional (2D) materials have shown tremendous potential in photovoltaic detectors and solar cells. However, such 2D photovoltaic devices are limited by low quantum efficiencies due to the severe interface recombination and the inefficient contacts. Here, we report an efficient MoS_2_/AsP vdW hetero-photodiode utilizing a unilateral depletion region band design and a narrow bandgap AsP as an effective carrier selective contact. The unilateral depletion region is verified via both the Fermi level and the infrared response measurements. The device demonstrates a pronounced photovoltaic behavior with a short-circuit current of 1.3 μA and a large open-circuit voltage of 0.61 V under visible light illumination. Especially, a high external quantum efficiency of 71%, a record high power conversion efficiency of 9% and a fast response time of 9 μs are achieved. Our work suggests an effective scheme to design high-performance photovoltaic devices assembled by 2D materials.

## Abstract (web summary)

Photovoltaic devices based on 2D materials still suffer from low quantum efficiencies due to interfacial charge recombination and inefficient contacts. Here, the authors design photovoltaic detectors and photodiodes based on MoS_2_ and doped AsP heterojunction with unilateral depletion region reporting high external quantum efficiency of 71% under zero applied bias.

## Introduction

Photodetectors based on two-dimensional (2D) layered materials have been widely studied since the discovery of graphene due to their atomic layer thicknesses, strong light-matter interactions and layer-dependent electronic and optical properties^1–8^. A variety of high-performance photodetectors with ultrahigh responsivity or gain, ultrahigh detectivity have been reported based on transition metal dichalcogenides (TMDs)^9–11^, III–VI group layered semiconductors^12–14^, black phosphorus^15,16^, and so on^17,18^, from visible to mid-infrared wavelength range^19–22^. Thus far, these 2D photodetectors with high responsivity are operated in photoconductive mode with high gain (including photogating mode), which suffer from either a large dark current or a slow response speed. Further, a bias is required for the device operation resulting in a static power dissipation, unsuitable for self-powered or portable battery-powered devices. A good photodetector should currently possess a high responsivity, a short response time, a high signal-to-noise ratio, and low or no power consumption for practical applications^23^. Photodiode detectors are the ideal choice for satisfying the above requirements. 2D-materials-based van der Waals heterostructures (vdWHs) offer an innovative avenue for this photodetection via the photovoltaic effect^24–26^. As compared with the conventional covalently or ionic bonded heterostructures, vdWHs possess an obvious advantage in terms of dangling bond-free interfaces that are free of lattice matching constraints, which opens up opportunities to exploit the numerous more combinations of materials with unique and widely disparate properties in a single vdWHs device^27^. Thus far, many artificial vdWHs aiming at photovoltaic detectors or solar cells have been assembled via the layer stacking or van der Waals epitaxy techniques^24,26^. Lee et al. reported the gate-tunable photovoltaic response in the atomically thin van der Waals p-n heterodiodes assembled by monolayer or multilayer n-type MoS_2_ and p-type WSe_2_, where an external quantum efficiency (EQE) of 34% was measured for multilayer p-n junctions^28^. Similar works were also reported independently by Cheng et al. and Furchi et al. at the same time^29,30^. In addition to WSe_2_/MoS_2_ vdWHs, various vertical vdWHs based on other 2D layered materials, such as BP/MoS_2_^31,32^, MoTe_2_/MoS_2_^33,34^, GaTe/MoS_2_^35,36^, GaTe/InSe^37^, GaSe/GaSb^38^, and AsP/InSe^39^, have also been explored in photovoltaic detectors and photovoltaics. However, the EQE (or power conversion efficiency) of those 2D heterodiodes is still low, most below 55% (5%) due to the severe tunneling-assisted interface recombination of the photogenerated electron-hole pairs in such thin bilateral depletion regions as well as the inefficient carrier selective contacts because of the Schottky barrier at the semiconductor-metal interface^4,24,28^. In addition, in these heterodiodes with bilateral depletion regions, both the photogenerated electrons and holes need to cross the heterointerface where they are easily trapped, leading to a slow response speed^34,35^. To resolve these problems, we propose and design a heterodiode with a unilateral depletion region. The unilateral depletion region heterodiode is defined as a heterojunction where one side near the heterointerface is carrier-depleted while the other side is carrier-accumulated (Supplementary Fig. 1 and Note 1). Based on this classification of heterojunctions, a pp or nn heterojunction can achieve this kind of heterodiode with a unilateral depletion region. In this proposed heterodiode, a narrow bandgap highly doped 2D semiconductor is used to form the type-I heterojunction and served as an effective carrier selective contact without any heterointerface barrier for photogenerated electrons or holes. In this case, only one type of photogenerated carriers in the depletion region, electrons or holes, cross the heterointerface and collected by the narrow bandgap 2D semiconductor via the rapid recombination with the opposite carriers in the accumulation region. Thus, the interface recombination of the photogenerated electron-hole pairs could be significantly suppressed and the response speed could be greatly promoted.

In this work, we choose molybdenum disulfide (MoS_2_) and black arsenic phosphorus (AsP) 2D layered semiconductors to construct the van der Waals heterodiode for realizing the unilateral depletion region to reduce the severe tunneling-assisted interface recombination and interface trapping effect. The elemental ratio of As to P in AsP alloy is about 1 ± 0.01 according to the element analysis in our previous work^39^. MoS_2_ is a promising 2D material for high-performance visible photodetectors^9,11^, and AsP is a typical p-type 2D semiconductor with a narrow bandgap of 0.2–0.3 eV in bulk^19,20,40^. Thick MoS_2_ flakes with weak p-type conduction and thick AsP flakes are exfoliated to build the van der Waals pp^+^ heterodiode. The MoS_2_/AsP heterodiode exhibits a gate-tunable backward-like diode behavior with a current rectification ratio over 6 × 10^3^. Upon 520 nm laser illumination, the heterodiode exhibits a pronounced photovoltaic effect with a short-circuit current as high as 1.3 μA and a large open-circuit voltage of 0.61 V, enabling an ultrahigh photocurrent to dark current ratio over 1 × 10^6^. Specifically, a high responsivity of 0.3 A W^−1^ and a high external quantum efficiency of 71% are obtained under zero bias. Remarkably, a high power conversion efficiency of 9% and a fast response time of 9 μs are achieved, which, to the best of our knowledge, are the highest values ever reported for photovoltaic detectors based on 2D vdWHs^24,25^.

## Results

### Device characterizations and electrical properties

**Fig. 1:**
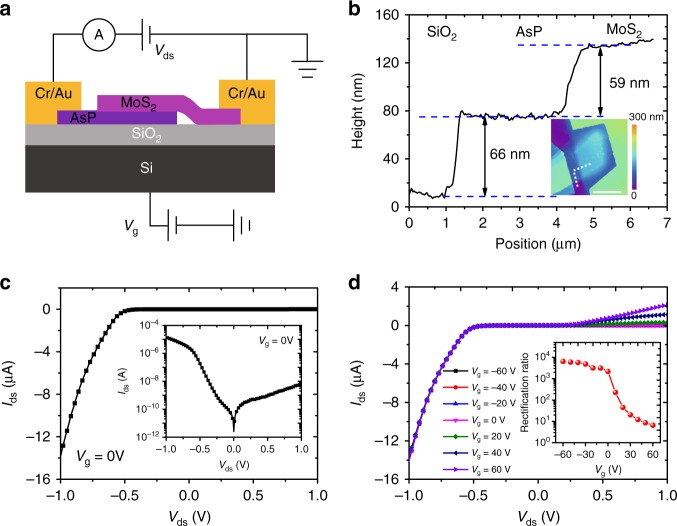
Electrical characterizations of the MoS_2_/AsP vdWHs device. **a** Schematic of the fabricated MoS_2_/AsP vdWHs device for electrical measurement. **b** Line scan profile of the heterostructure from the AFM image shown in the inset. Scale bar is 5 μm. **c** *I*_ds_–*V*_ds_ curve of the MoS_2_/AsP vdWHs device at gate voltage *V*_g_ = 0 V. The inset is the *I*_ds_–*V*_ds_ curve in semi-logarithmic scale. **d** *I*_ds_–*V*_ds_ curves of the MoS_2_/AsP vdWHs device at different gate voltages varying from −60 to 60 V. The inset is the gate-dependent rectification ratios of the MoS_2_/AsP vdWHs device

Figure 1a shows the schematic of the fabricated MoS_2_/AsP vdWHs device. Immediately after the fabrication process, the device was coated by a thin PMMA layer to protect it from oxidation by oxygen in the air. The MoS_2_ flake is placed on the AsP flake for better light absorption owing to a much larger bandgap value. The heavily p-doped Si substrate is used as a back gate to tune the carrier densities in each flake. The metal on the AsP side is defined as drain electrode while that on MoS_2_ side as source electrode. Supplementary Fig. 2a presents the optical image of the completely fabricated MoS_2_/AsP vdWHs device, where we can see both the MoS_2_ and AsP flakes are relatively thick. In order to investigate the properties of the individual MoS_2_, AsP flakes and the heterodiode separately, each of the MoS_2_ and AsP flakes are wired with two electrodes. Figure 1b shows the line scan profile of the heterostructure from the atomic force microscope (AFM) image, and the inset is the AFM image of the fabricated device. No air bubbles except for some surface contaminant can be seen in overlapping heterojunction region, indicating a good interface quality of the heterostructure. The thicknesses of MoS_2_ and AsP flakes are estimated to be 66 and 59 nm, respectively. The crystalline quality of MoS_2_, AsP and heterostructure were examined by Raman characterization (Supplementary Fig. 2b). Two strong peaks are observed at 385 and 410.5 cm^−1^ in the Raman spectrum of MoS_2_ flake, representing the in-plane vibration mode $E_{2{\mathrm{g}}}^1$ and out-of-plane vibration mode $A_{1{\mathrm{g}}}$, respectively^33^. For AsP flake, three main peaks are located at 226, 236, and 252 cm^−1^, corresponding to the $A_{\mathrm{g}}^1$, $B_{\mathrm{g}}^2$, and $A_{\mathrm{g}}^2$ modes, respectively^19^. No peak shift or degradation is observed in the spectrum of the heterojunction region compared to the peaks from the individual MoS_2_ flake, indicating the high crystalline quality of the junction region. The Raman peaks of the AsP component in the junction region almost disappears, most likely due to limited input light reaching the AsP flake owing to the thick MoS_2_.

The electrical characteristics of the MoS_2_/AsP vdWHs device are measured first in an ambient environment. Interestingly, a large drain current over 10 μA at negative bias and a small current of several nA at positive bias are observed in Fig. 1c, indicating a backward-like diode is formed, which is contrary to one’s expectations at first glance. Since few layer MoS_2_ (with thickness of several nanometers) is a typical n-type 2D semiconductor and AsP is an intrinsically p-type, a normal pn junction forward diode is often observed when these two materials are stacked to build the heterojunction. To understand the mechanism of this contrary electrical behavior of the MoS_2_/AsP vdWHs diode, the electrical transport of both the individual MoS_2_ and AsP field effect transistors (FET) are separately measured. As shown in Supplementary Fig. 3, the transfer curve of the thick MoS_2_ FET exhibits an abnormal light p-type conduction behavior with a very weak gate modulation of the channel carrier density. The p-type conduction of thick MoS_2_ flakes (with thickness larger than 50 nm) is further verified via a systematic investigation of MoS_2_ FETs with different flake thicknesses (Supplementary Fig. 15 and Note 4). The light p-type conduction of thick MoS_2_ here may be attributed to the impurities-induced doping effect or the defect-induced doping effect (e.g., S adatoms)^41,42^. The weak gate control is likely due to the thick MoS_2_ channel. In addition, the nonlinear *I*–*V* curves of MoS_2_ FET indicate a Schottky contact between Cr metal and MoS_2_. The transfer curve of the AsP FET (Supplementary Fig. 4a) shows a large channel current and strong p-type conduction over the gate voltage range from −60 to 60 V with mild modulations, which means the Fermi level is close to the valance band maximum of AsP and the hole density is high. The linear *I*–*V* curves of AsP FET (Supplementary Fig. 4b, c) indicate an Ohmic contact between Cr metal and AsP. Based on these results, we can conclude that a pp^+^ heterojunction is formed in this MoS_2_/AsP vdWHs diode. The Fermi level position is vital in the operation of this backward-like diode. The gate-tunable *I*–*V* curves of the MoS_2_/AsP vdWHs diode are also measured and shown in Fig. 1d and Supplementary Fig. 5. It is interesting that the positive current monotonically increases while the negative current is almost unchanged when sweeping *V*_g_ from −60 to 60 V. This suggests that the gate modulation of diode positive current results from the modulation of hole concentration in AsP, which influences the probability of hole tunneling through the heterojunction. The gate-dependent rectification ratio is shown in the inset in Fig. 1d. A high rectification ratio over 6 × 10^3^ is obtained at *V*_g_ = −60 V. The rectification ratio decreases fast at the negative *V*_g_ and then saturates near to 1 at the positive *V*_g_ with increasing *V*_g_, showing a gate-tunable current rectifying of the MoS_2_/AsP vdWHs diode.

### Device operation mechanism and verification of unilateral depletion region

**Fig. 2:**
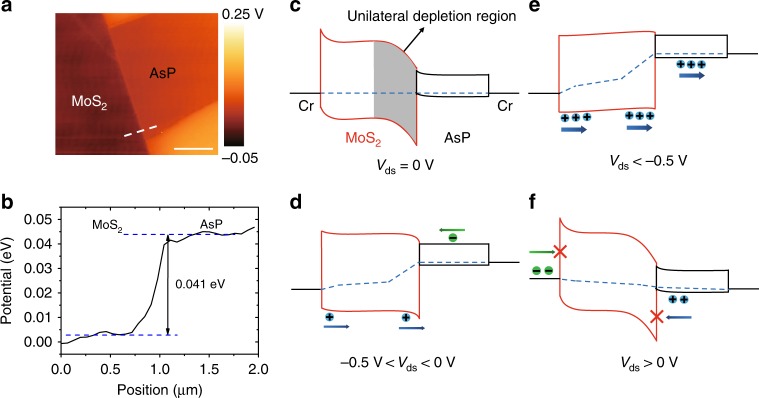
Kelvin probe force microscopy (KPFM) characterization and energy band alignments of the MoS_2_/AsP vdWHs diode. **a** Surface potential image of a clean MoS_2_/AsP heterostructure with similar layer thicknesses. Scale bar is 2 μm. **b** Potential line profile across the heterostructure edge showing the Fermi level difference between MoS_2_ and AsP. The corresponding energy band alignments of the MoS_2_/AsP vdWHs diode at different bias, **c** *V*_ds_ = 0 V, **d** −0.5 V < *V*_ds_ < 0 V, **e** *V*_ds_ < −0.5 V, and **f** *V*_ds_ > 0 V

As above state, the relative position of Fermi level determines the operation of this pp^+^ heterojunction. Thus, kelvin probe force microscopy (KPFM) technique is used to measure the surface potential of both layers, from which the Fermi level difference can be obtained. To accurately obtain the surface potential, we assembly a clean MoS_2_/AsP heterostructure with the similar layer thicknesses (Supplementary Fig. 6). As shown in Fig. 2a, b, the Fermi level of AsP is ~41 meV higher than that of MoS_2_. Based on these results and the band structures of thick MoS_2_ and AsP layers (Supplementary Fig. 7)^40,43^, the energy band alignments of the MoS_2_/AsP vdWHs diode at different bias conditions are depicted in Fig. 2c–f. In equilibrium state, after MoS_2_ and AsP are brought in physical contact with each other, the minority electrons in AsP will move into MoS_2_, creating more holes in AsP. As a result, a hole accumulation region is formed at AsP side whereas a wide depletion region formed at the MoS_2_ side. In other words, a heterojunction with unilateral depletion region in MoS_2_ is formed. At small negative bias −0.5 V < *V*_ds_ < 0 V, holes injected from the MoS_2_ side are hindered by both the large heterojunction barrier and the Schottky barrier, while minority electrons are injected from the AsP side, thereby leading to a small current magnitude as shown in Fig. 1c. At large negative bias *V*_ds_ < −0.5 V, both the heterojunction barrier and the Schottky barrier are reduced. Thus, holes are easily injected into MoS_2_ and then cross over the heterojunction barrier, forming a large hole-dominant current as shown in Fig. 1c. At positive bias *V*_ds_ > 0 V, although the holes are easily injected into AsP, they are blocked by the large valance band offset (~0.8 eV) at the MoS_2_/AsP interface. Meanwhile the electrons can hardly be injected into MoS_2_ because of the large Schottky barrier for electrons. Accordingly, the current is low at positive bias as shown in Fig. 1c.

**Fig. 3:**
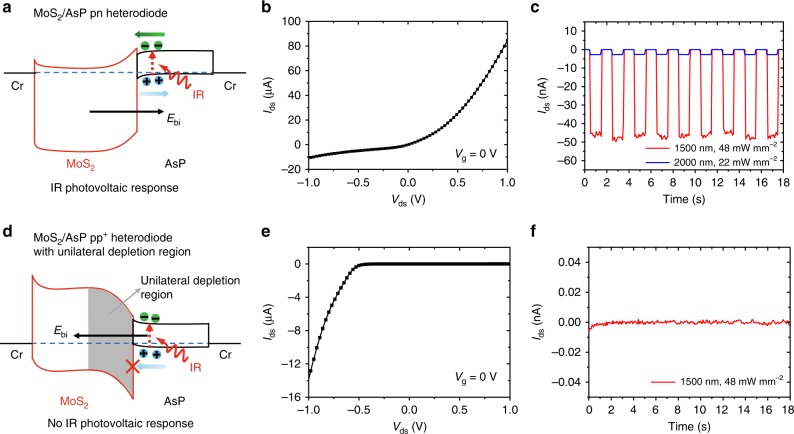
Distinction of the different band structures of the MoS_2_/AsP pn and pp+ heterodiodes. Energy band diagrams of the **a** MoS_2_/AsP pn heterodiode and **d** MoS_2_/AsP pp^+^ heterodiode under IR laser illumination at zero bias. *I*_ds_–*V*_ds_ curves of the **b** MoS_2_/AsP pn heterodiode and **e** MoS_2_/AsP pp^+^ heterodiode. **c** IR (1500 and 2000 nm) photovoltaic response of the MoS_2_/AsP pn heterodiode. **f** IR (1500 nm) photovoltaic response of the MoS_2_/AsP pp^+^ heterodiode with unilateral depletion region

To further confirm the unilateral depletion region is formed in this MoS_2_/AsP vdWHs pp^+^ heterodiode, an electrical *I*–*V* curve and infrared (IR) photovoltaic response method are used to distinguish the different band structures of the pp^+^ and pn heterojunctions. A normal MoS_2_/AsP pn heterodiode is fabricated as a control by choosing a thin MoS_2_ (thickness of 9 nm) and AsP (thickness of 21 nm) flakes (Supplementary Fig. 16). As shown in Fig. 3a, for the normal MoS_2_/AsP pn heterodiode, both the n-type MoS_2_ and p-type AsP are partially depleted (bilateral depletion region), resulting in a built-in electric field with direction pointing to AsP side. Therefore, a regular forward diode behavior is observed, as shown in Fig. 3b. When an IR light is incident, the photogenerated electrons in AsP can easily cross over the heterointerface due to the small conduction band offset, thus a negative short-circuit current is formed in this normal MoS_2_/AsP pn heterodiode, as shown in Fig. 3c. In contrast; in the MoS_2_/AsP pp^+^ heterodiode with unilateral depletion region, since the MoS_2_ is hole-depleted and AsP is hole-accumulated, the direction of the built-in electric field is pointing towards the MoS_2_ side, opposite to that in MoS_2_/AsP pn heterodiode. Therefore, an abnormal backward-like diode behavior is observed, as shown in Fig. 3e. Under IR illumination, the photogenerated holes are blocked by the large valance band offset, resulting in no IR photovoltaic response, as shown in Fig. 3f. Both the *I*–*V* curves and the IR photovoltaic response definitely confirm that our MoS_2_/AsP pp^+^ heterodiode has a unilateral depletion region in its band structure.

### High efficiency and fast photovoltaic response of the MoS_2_/AsP vdWHs diode

**Fig. 4:**
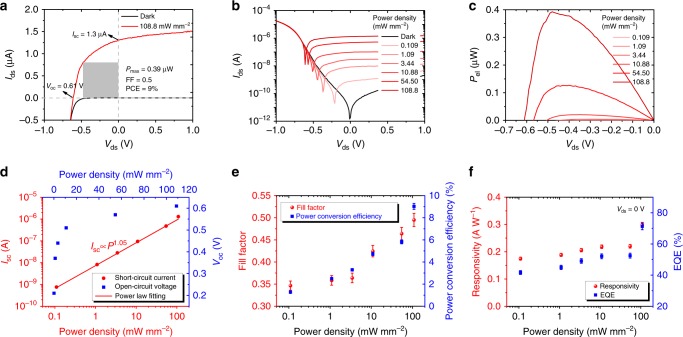
Photovoltaic response of the MoS_2_/AsP vdWHs diode. **a** *I*_ds_–*V*_ds_ curves of the MoS_2_/AsP vdWHs diode under dark (black line) and 520 nm laser illumination (red line), respectively. The gray region indicates where the maximum electrical power is obtained. **b** *I*_ds_–*V*_ds_ curves of the MoS_2_/AsP vdWHs diode under 520 nm laser illumination with different power densities. **c** Output electrical power *P*_el_ as a function of *V*_ds_. **d** Power dependent short-circuit current *I*_sc_ and open-circuit voltage *V*_oc_. **e** Power dependent fill factor (FF) and power conversion efficiency (PCE). **f** Power dependent responsivity and external quantum efficiency (EQE). Error bars represent standard deviation

Next, we focus on the photoresponse properties of the MoS_2_/AsP vdWHs diode with unilateral depletion region and evaluate its performance in photovoltaic detectors in visible wavelength range. All the photoresponse measurements are conducted at zero gate voltage (*V*_g_ = 0 V) unless otherwise stated. Figure 4a shows the *I*_ds_–*V*_ds_ curves of the MoS_2_/AsP vdWHs diode under dark and 520 nm laser illumination, respectively. Obviously, the curve is upshifted under illumination, exhibiting a distinct photovoltaic response. A high short-circuit current *I*_sc_ of 1.3 μA and a large open-circuit voltage *V*_oc_ of 0.61 V are obtained under the power density of 108.8 mW mm^−2^ corresponding to 100× AM 1.5 intensity. The photocurrent to dark current ratio is over 1 × 10^6^ at zero bias, showing an ultrahigh signal-to-noise ratio. As a comparison, no obvious visible photovoltaic response is observed in the normal MoS_2_/AsP pn heterodiode (Supplementary Figs. 16–19). The output electrical power *P*_el_, which describes the electricity generation capability of a photovoltaic detector or solar cell, is defined as *P*_el_ = *I*_ds_·*V*_ds_. The gray rectangle region in Fig. 4a indicates where the maximum electrical power *P*_max_ can be obtained. The fill factor (FF) and power conversion efficiency (PCE) can be calculated as follows: FF = *P*_max_/*I*_sc_·*V*_oc_, PCE = *P*_max_/*P*_in_, where *P*_in_ is the incident optical power^25^. A *P*_max_ of 0.39 μW, a FF of 0.5 and a PCE of 9% are achieved under the power density of 108.8 mW mm^−2^ or incident power of 4.35 μW. The large PCE, to the best of our knowledge, is the highest value ever reported for photovoltaic detectors based on 2D vdWHs^24,25^. We are aware that absorption efficiency is thickness-dependent, thus the quantum efficiency and power conversion efficiency are thickness-related. To investigate the effect of thickness on the device performance, six MoS_2_/AsP heterojunction devices with different layer thicknesses (Supplementary Fig. 21) are fabricated according to the conduction type of MoS_2_ and AsP flakes, as classified in the Supplementary Table 1 and Note 5. Via comprehensive comparison of the photovoltaic response of the six devices (Supplementary Figs. 22–31 and Table 2), it can be concluded that the thickness-related absorption efficiency of MoS_2_ is not the major reason for the large performance variation. Instead, the thickness-dependent band profile of the heterojunction is the key factor determining the photovoltaic efficiency of the MoS_2_/AsP heterodiode, as detailed below.

To further investigate the photovoltaic response of the MoS_2_/AsP vdWHs diode, power dependent photoresponse are measured. As shown in Fig. 4b and Supplementary Fig. 8, with increasing the optical power, the *I*–*V* curves keep on shifting upward. Thus the output electrical power *P*_el_ are increased as shown in Fig. 4c. From Fig. 4d, the short-circuit current *I*_sc_ increases almost linearly with optical power (*I*_sc_ ∝ *P*^1.05^ by power law fitting), whereas open-circuit voltage *V*_oc_ shows a logarithmic relationship with optical power (Supplementary Fig. 14 and Note 3). Figure 4e displays the power dependent fill factor (FF) and power conversion efficiency (PCE). Both the FF and PCE decrease with decreasing incident power. Nevertheless, a PCE greater than 1% is still obtained at power density as low as 0.109 mW mm^−2^, corresponding to an incident power of 4.25 nW. To quantitatively evaluate the performance of the MoS_2_/AsP vdWHs photovoltaic detector, responsivity and external quantum efficiency (EQE) are calculated at zero drain bias. The responsivity is defined as *R* = *I*_ph_/*P*_in_·*A*, where *I*_ph_ is the net photocurrent, *A* is the junction area of the vdWHs device. EQE is the ratio of collected charge carriers to incident photons and can be calculated by EQE = *I*_sc_·*h*·*c*/*e*·*λ*·*P*_in_, where *h*, *c*, *λ* are the Planck’s constant, the speed of light and wavelength of the incident laser, respectively. A responsivity of 0.3 A W^−1^ and EQE of 71% are obtained at power density of 108.8 mW mm^−2^. Considering the large power variation range (three orders of magnitudes), the responsivity and EQE of the MoS_2_/AsP vdWHs photovoltaic detector is relatively robust, showing a good linear dynamic range. The gate modulated photovoltaic response of the MoS_2_/AsP vdWHs diode are also measured and provided in Supplementary Fig. 9 and Note 2.

**Fig. 5:**
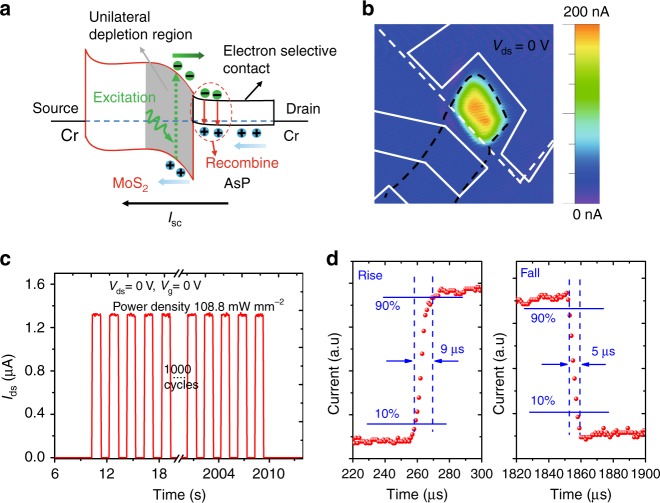
Photocurrent mapping and response time measurement of the MoS_2_/AsP vdWHs diode. **a** Energy band diagram of the MoS_2_/AsP vdWHs diode under 520 nm laser illumination at zero bias. **b** Scanning photocurrent mapping of the MoS_2_/AsP vdWHs diode under 520 nm laser illumination with the laser spot size less than 1 μm at *V*_ds_ = 0 V. **c** Photoswitching response of the MoS_2_/AsP vdWHs diode at *V*_ds_ = 0 V. **d** Time-resolved photoresponse of the MoS_2_/AsP vdWHs diode at *V*_ds_ = 0 V

The mechanism of the enhanced photovoltaic response of the device under 520 nm laser illumination can be explained by the energy band diagram of the MoS_2_/AsP vdWHs diode. As shown in Fig. 5a, when the device is under 520 nm illumination, most of the electron-hole pairs are generated in the top MoS_2_ channel due to the relatively thick MoS_2_ flake. Under the large built-in electric field in the MoS_2_ unilateral depletion region, the electrons freely move to the AsP side and are collected by the drain electrode, meanwhile the holes move to the MoS_2_ side and are collected by the source electrode. Thus, a negative *V*_oc_ and positive *I*_sc_ is observed in Fig. 4a. Note that only electrons cross the heterointerface without any barrier block and efficiently collected by the AsP carrier selective contact via the rapid recombination with majority holes in AsP not the photogenerated holes in the MoS_2_ unilateral depletion region. By this means, although a little part of energy (approximately 0.25 eV per one photon) is lost, the recombination of photogenerated electron-hole pairs are significantly suppressed and the carrier collection efficiency is greatly improved, thus the EQE and PCE are enhanced as we mentioned previously. In order to confirm the photovoltaic response is mainly induced by the heterojunction not the Schottky junction effect, scanning photocurrent mapping is used to acquire the photocurrent distribution. As seen in Fig. 5b, a clear photocurrent is observed in the whole junction area and no obvious current is generated near the metal contacts, which unambiguously demonstrates the photovoltaic current indeed originates from the heterojunction. We also demonstrate that no photovoltaic response is seen for the MoS_2_ FET device (Supplementary Fig. 13). The photoswitching and response speed are also important for a practical photodetector. Figure 5c presents the photoswitching response of the diode at zero bias under a periodical on/off laser illumination. The photoswitching response under different power densities and different biases are provided in Supplementary Figs. 11 and 12, respectively. The current rapidly increases when the laser is on and recovers to the dark state quickly when is off. A good stability is demonstrated with multiple and reproducible switching. The response time is achieved by using a digital oscilloscope to record the fast-varying photocurrent signals. As shown in Fig. 5d, the rise and decay processes are very fast with a rise time of 9 μs and a fall time of 5 μs, which are among the fastest response times for photovoltaic detectors based 2D vdWHs^29,34,44,45^. The fast response is attributed to the unilateral depletion region design, by which the interface trapping effect is greatly reduced since only electrons cross the interface and rapidly recombine with the holes in the accumulation region. This is confirmed by the response time of the normal MoS_2_/AsP pn heterodiode (Supplementary Fig. 20), which shows one order of magnitude slower response time than that of the MoS_2_/AsP heterodiode with unilateral depletion region. The spectral response of the MoS_2_/AsP vdWHs diode is also measured and shown in Supplementary Fig. 10. The diode shows a broadband photovoltaic response from visible to near infrared wavelengths with a peak response at about 600 nm, indicating its potential application in solar cells. Finally, for a comprehensive comparison, the figure of merits for photovoltaic detectors or solar cells based on 2D vdWHs are shown in Supplementary Table 3. It can be seen that our device based on thick MoS_2_/AsP vdWHs with unilateral depletion region manifests the highest PCE and fastest response time. The fill factor and open-circuit voltage *V*_oc_ are also comparable to previous works based on other vertical 2D vdWHs diodes. Our work suggests van der Waals heterodiodes based on 2D materials with unilateral depletion region band designs and narrow bandgap semiconductor carrier selective contacts are more suitable for high-performance photovoltaic detectors and solar cells.

### MoS_2_/BP vdWHs photodiode with unilateral depletion region

The vdWHs photodiodes with unilateral depletion region band structure can be applied to other 2D semiconductors, for example BP (similar to AsP). A MoS_2_/BP vdWHs photodiode with unilateral depletion region was successfully fabricated and demonstrated similar photovoltaic response (Supplementary Figs. 32–35 and Note 6). In comparison with MoS_2_/AsP vdWHs photodiode, the MoS_2_/BP vdWHs photodiode exhibits lower photovoltaic efficiency and device performance, which may be attributed to a different band alignment of the heterojunction and/or the unoptimized band profile of the device.

## Discussions

In summary, we have proposed and demonstrated a van der Waals heterodiode with a unilateral depletion region and a narrow bandgap semiconductor carrier selective contact for high-performance photovoltaic detectors, which was successfully fabricated by assembling thick weak p-type MoS_2_ and narrow bandgap AsP 2D layers. We show that this kind van der Waals heterodiode can overcome the severe tunneling-assisted interface recombination of photogenerated electron-hole pairs and inefficient carrier selective contacts inevitable in previous 2D heterodiodes based photovoltaic detectors. Meanwhile the unilateral depletion region configuration can greatly reduce the interface trapping effect of the photogenerated carriers. As a result, the MoS_2_/AsP heterodiode exhibits a pronounced photovoltaic effect with a short-circuit current as high as 1.3 μA and a large open-circuit voltage of 0.61 V under 520 nm laser illumination. More importantly, a record high power conversion efficiency of 9% and a fast response time of 9 μs are achieved. Our work lays a foundation for the next generation of 2D vdWHs photovoltaic devices comprising a combination of a large bandgap semiconductor as the depleted absorption layer and a narrow bandgap semiconductor as the effective carrier selective contact.

## Methods

### Device fabrication

The MoS_2_/AsP vdWHs investigated here were fabricated by a commonly used dry transfer method utilizing a polydimethylsiloxane (PDMS) carrier. First, relative thick AsP flakes were mechanically exfoliated from an as-grown AsP crystal using Scotch tape and transferred onto a silicon substrate (with 300 nm SiO_2_). Then, MoS_2_ flakes were exfoliated onto the PDMS film from the commercial bulk crystal (2D Semiconductors, America) and transferred onto the AsP flake to form the heterostructure under the optical microscope assisted by an aligned transfer platform. The exfoliation and transfer processes were conducted in the N_2_-protection glovebox to minimize the oxidation of AsP. For device fabrication, electron-beam lithography (EBL, FEI F50 SEM with NPGS system) was employed to define the electrode patterns. A Cr/Au (15 nm/55 nm) metal thin film was deposited as the contact electrodes by thermal evaporation, followed by the standard lift-off process.

### Characterizations and measurements

Raman spectra were achieved by a confocal Raman/PL system (LabRAM HR800) using 532 nm laser as an excitation source. The thickness and surface potential of the two layers were obtained by AFM and KPFM, respectively (Bruker Multimode 8). The electrical characterizations of the MoS_2_/AsP vdWHs devices were performed in a probe station (Lake Shore) using a semiconductor device parameter analyzer (Agilent, B1500). The devices were wire-bonded onto a 24-pin chip carrier and a 520 nm laser was used for photoresponse measurements. A supercontinuum light source (YSL sc-pro) is employed to obtain the spectral photocurrent response. An oscilloscope (Tektronix DPO 5204, 100 MHz, 2.5 G/s) was used to record the transient photocurrent response to resolve the photoresponse time. In all the photocurrent measurements, the laser was focused on the device with a ×20 objective (NA = 0.45), and the laser spot is near flat with a size of about ∼20 μm, larger than the heterojunction area to make sure a uniform illumination. All the devices were measured at room temperature in an ambient environment.

## Supplementary information

Supplementary Information

Source Data

## Data Availability

The data that support the plots within this paper and other findings of this study are available from the corresponding author upon reasonable request. The source data underlying Figs. 1a, c, 3b, c, e, f, 4a–f, 5c, d and Supplementary Figs. 2b, 3–5, 8–13, 15, 17–20, 22–27, 29, 30, and 32–35 are provided as a Source Data file.
