# Supplementary material for: High efficiency and fast van der Waals hetero-photodiodes with a unilateral depletion region
Source: Nat Commun. 2019 Oct 11;10:4663. doi: 10.1038/s41467-019-12707-3 (PMC6789142; doi:10.1038/s41467-019-12707-3)
Supplement: Supplementary file 1 — Supplementary Information [file 41467_2019_12707_MOESM1_ESM.pdf]

Supplementary Information

High efficiency and fast van der Waals hetero-photodiodes with a unilateral depletion region

Feng Wu et al.

Supplementary Figures

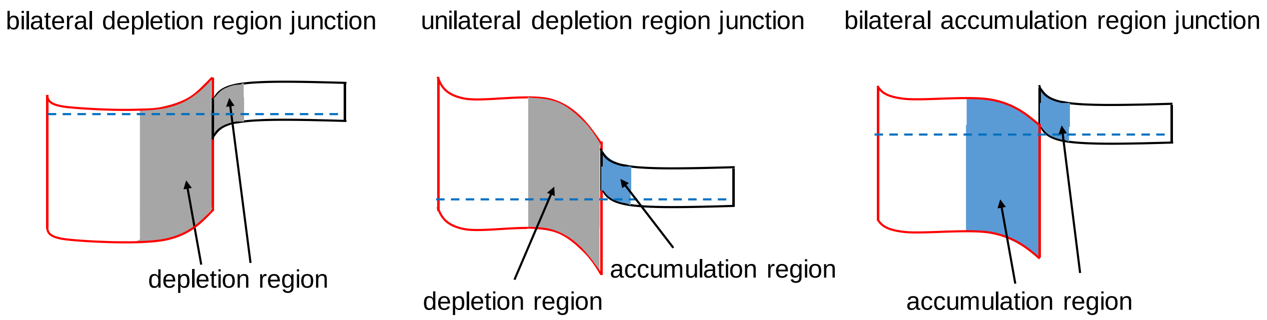

Supplementary Figure 1. Schematic band profiles of the heterojunctions showing bilateral depletion region, unilateral depletion region and bilateral accumulation region junctions.

MoS_2/AsP vdWHs photodiode with unilateral depletion region

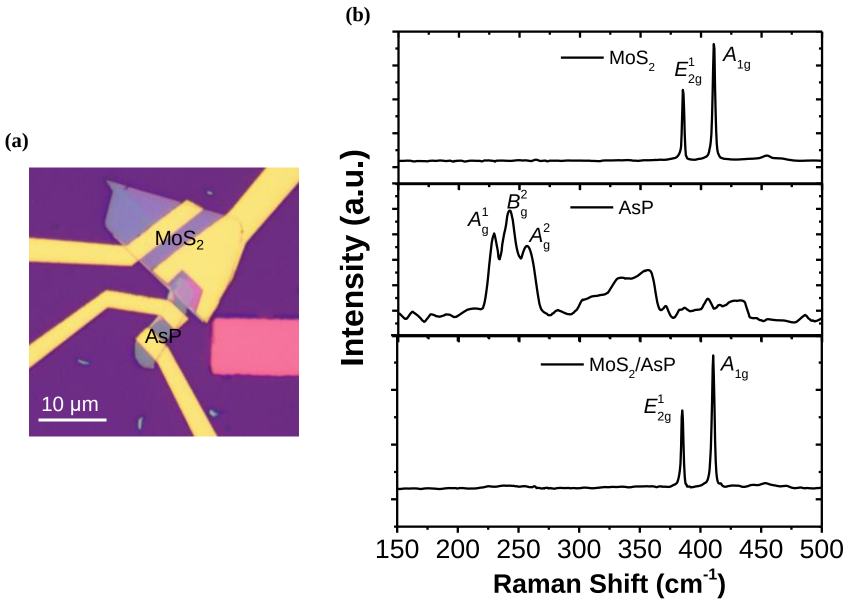

Supplementary Figure 2. (a) Optical image of the fabricated MoS_2/AsP vdWHs device. (b) Raman spectra of the individual MoS_2, AsP flakes and MoS_2/AsP heterostructure.

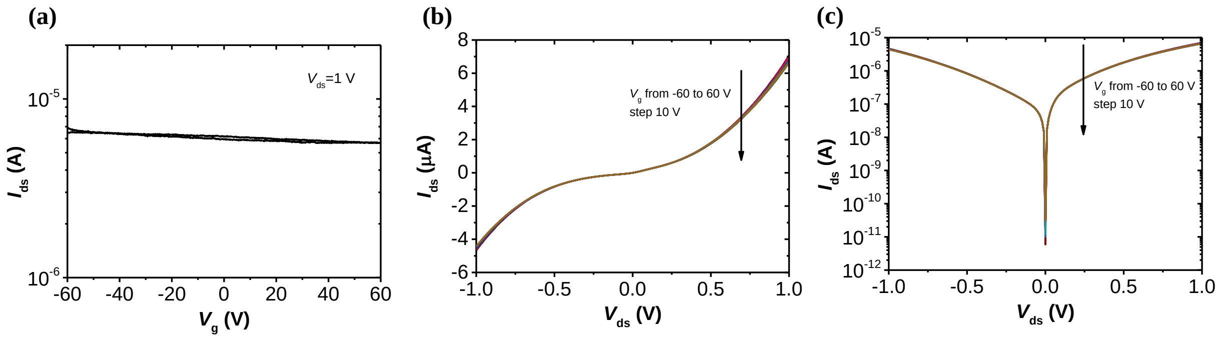

Supplementary Figure 3. Electrical characterizations of individual MoS_2 FET. (a) Transfer curve of MoS_2 FET. I_{ds}-V_{ds} curves of MoS_2 FET in (b) linear and (c) semi-logarithmic scale.

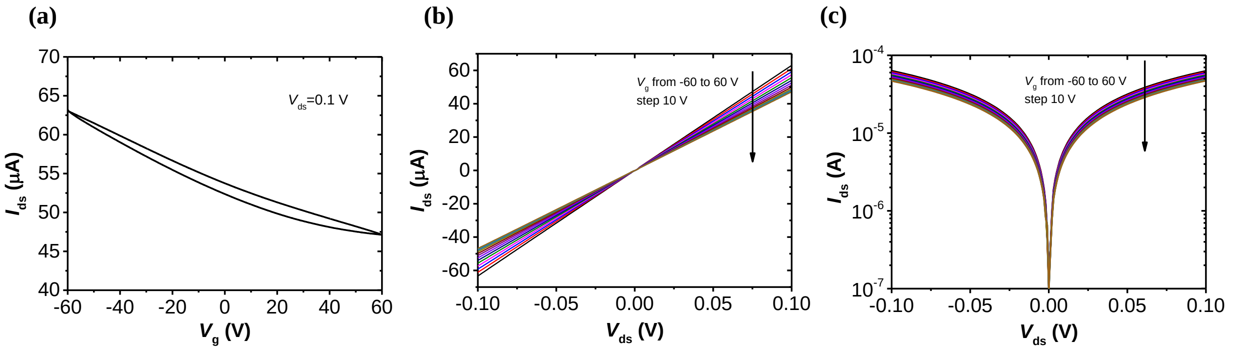

Supplementary Figure 4. Electrical characterizations of individual AsP FET. (a) Transfer curve of AsP FET. I_{ds}-V_{ds} curves of AsP FET in (b) linear and (c) semi-logarithmic scale.

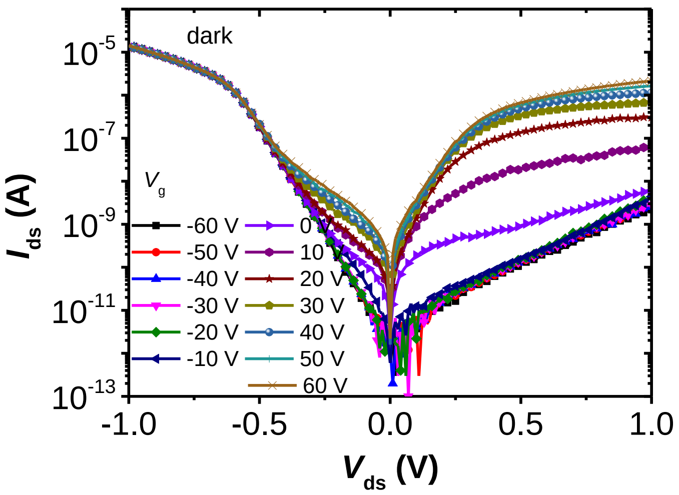

Supplementary Figure 5. I_{ds}-V_{ds} curves of MoS_2/AsP vdWHs diode in semi-logarithmic scale.

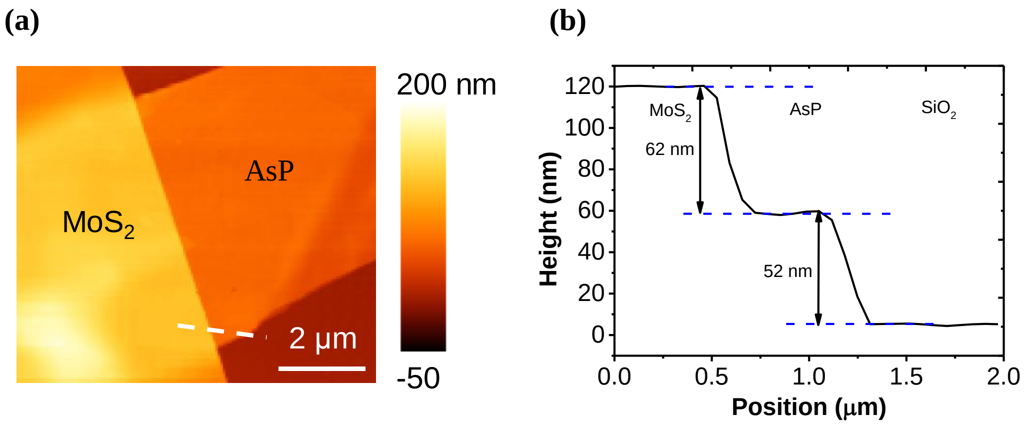

Supplementary Figure 6. AFM characterization of a clean MoS_2/AsP vdWHs with similar layer thicknesses. (a) AFM image. (b) Height line profile.

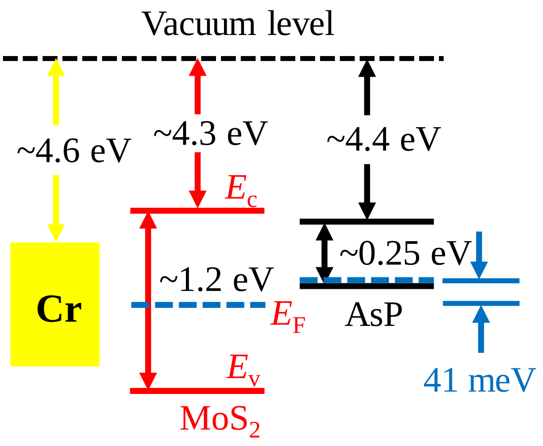

Supplementary Figure 7. Energy band profiles of Cr, MoS_2 and AsP prior to contact, showing the Fermi level of AsP is 41 meV higher than that of MoS_2.

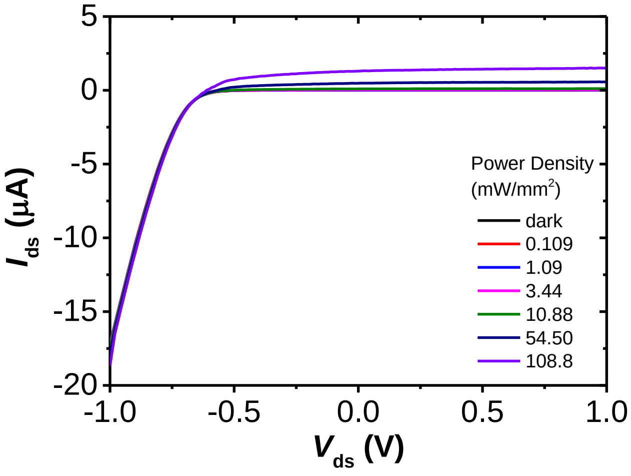

Supplementary Figure 8. I_{ds}-V_{ds} curves of MoS_2/AsP vdWHs diode under 520 nm laser illumination with different power densities in linear scale.

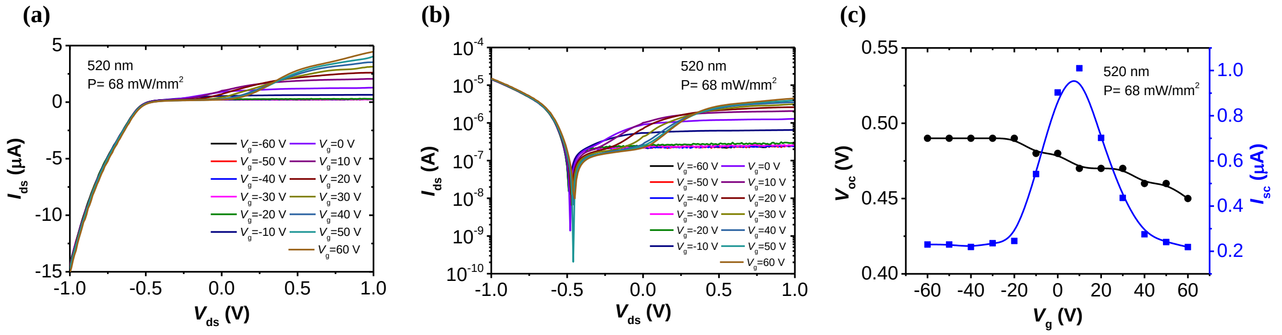

Supplementary Figure 9. Gate-dependent photovoltaic response of MoS_2/AsP vdWHs diode under 520 nm laser illumination. (a) I_{ds}-V_{ds} curves of MoS_2/AsP diode under different gate voltages in linear scale. (b) I_{ds}-V_{ds} curves of MoS_2/AsP diode under different gate voltages in semi-logarithmic scale. (c) Gate voltage dependence of open-circuit voltage V_{oc} and short-circuit current I_{sc}.

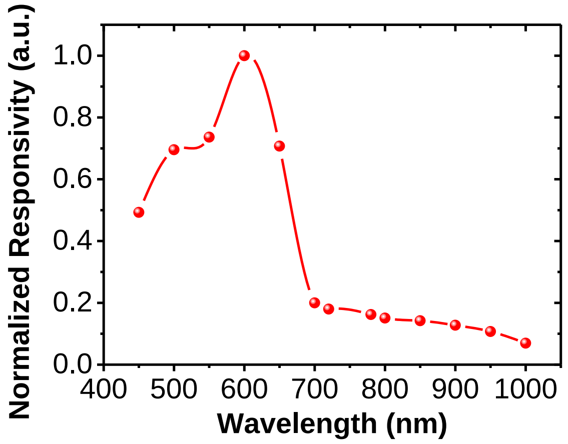

Supplementary Figure 10. Spectral response of the MoS_2/AsP vdWHs diode at V_{ds} =0 V.

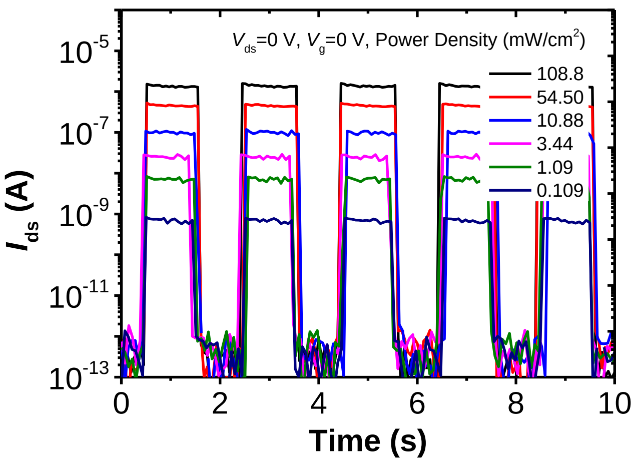

Supplementary Figure 11. Photoswitching response of the MoS_2/AsP vdWHs diode under 520 nm laser illumination with different power densities

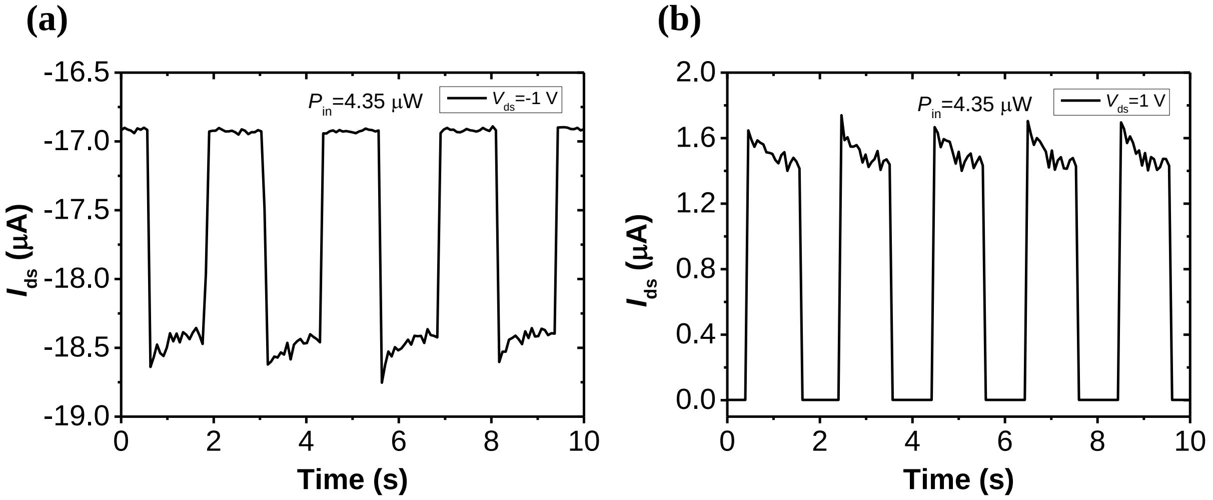

Supplementary Figure 12. Photoresponse of the MoS_2/AsP vdWHs diode under 520 nm laser illumination at (a) V_{ds}=-1 V and (b) V_{ds}=1 V.

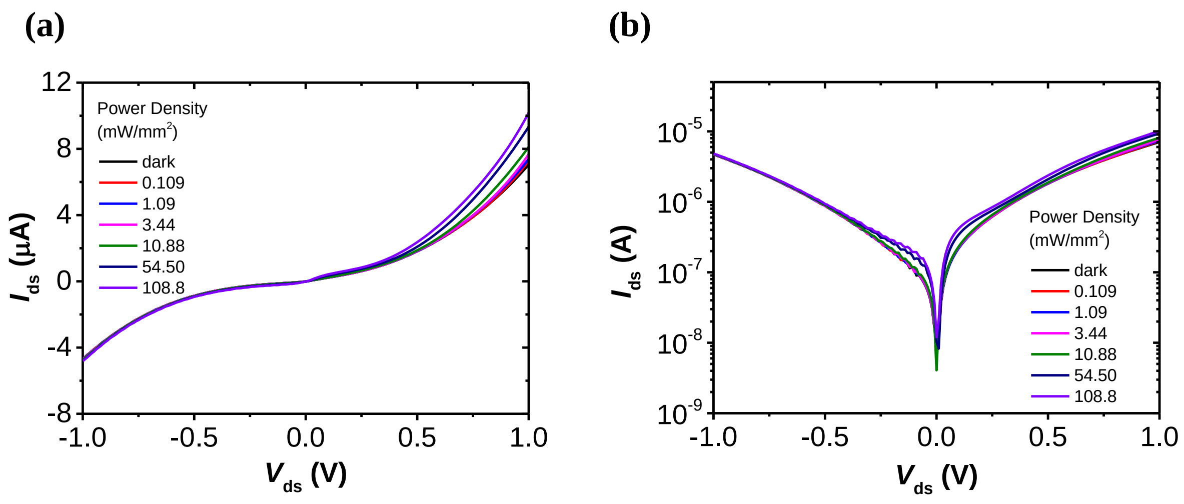

Supplementary Figure 13. I_{ds}-V_{ds} curves of MoS_2 FET under 520 nm laser illumination with different power densities in (a) linear scale and (b) semi-logarithmic scale.

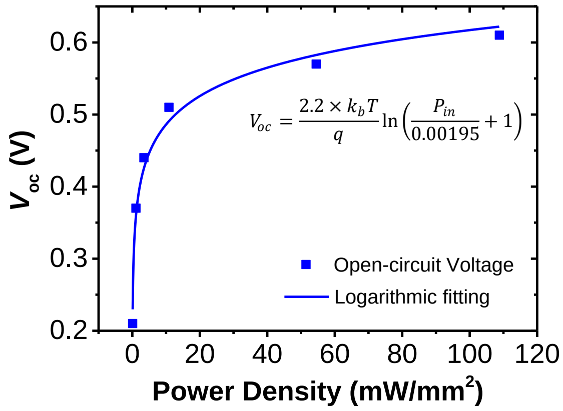

Supplementary Figure 14. Power dependent open-circuit voltage V_{oc}, solid symbol is experimental data and solid curve is the logarithmic fitting.

Thickness dependent conduction type of MoS_2 flake

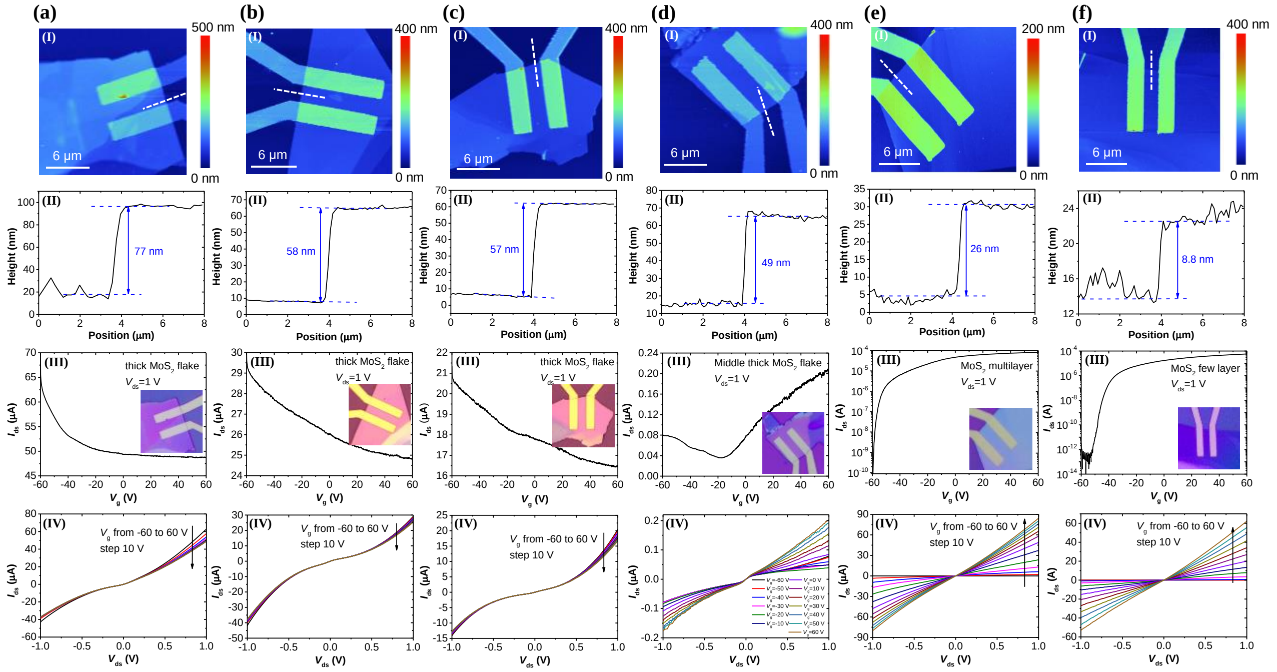

Supplementary Figure 15. (a)-(f) AFM images (I), corresponding thickness profiles (II), transfer curves (II) and output curves (IV) of MoS_2 FETs with different thicknesses, showing the conduction type changing from p-type to bipolar and finally n-type when the thickness of MoS_2 flake decreases.

Normal MoS_2/AsP pn heterodiode and its photoresponse

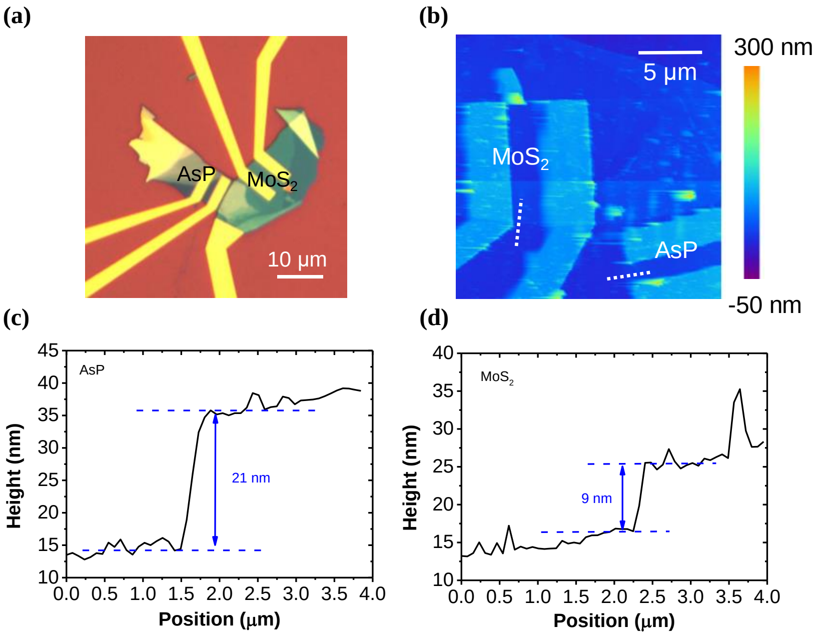

Supplementary Figure 16. Characterizations of the normal MoS_2/AsP pn heterodiode. (a) Optical image of the device. (b) AFM image of the device. Corresponding thickness profiles of (c) AsP and (d) MoS_2 layers.

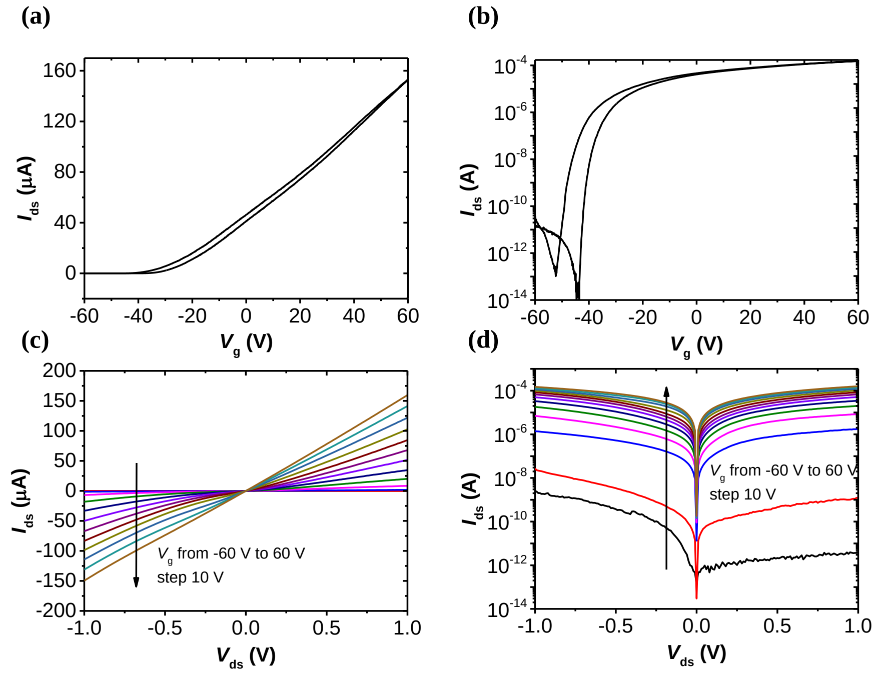

Supplementary Figure 17. Electrical characterizations of individual MoS_2 FET. Transfer curve of the MoS_2 FET at V_{ds}=1 V in (a) linear scale and (b) semi-logarithmic scale. I_{ds}-V_{ds} curves of the MoS_2 FET under different gate voltages in (c) linear scale and (d) semi-logarithmic scale.

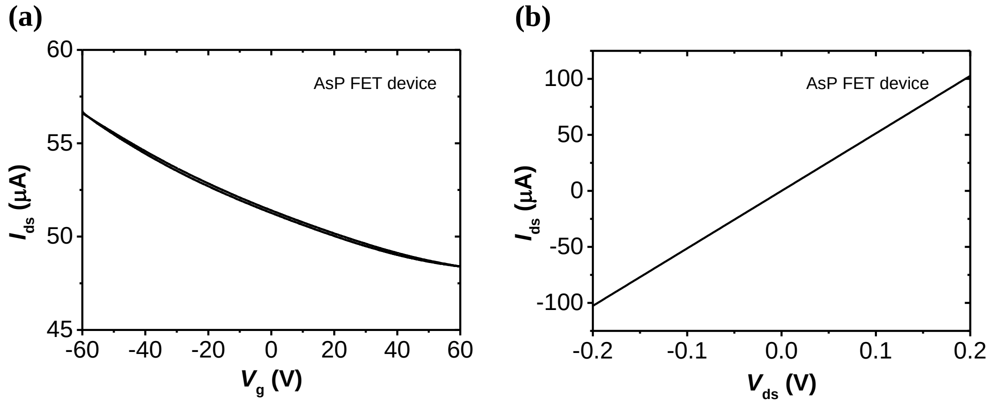

Supplementary Figure 18. Electrical characterizations of individual AsP FET. (a) Transfer curve of the AsP FET at V_{ds}=0.1 V. (b) I_{ds}-V_{ds} curve of the AsP FET at V_g=0 V.

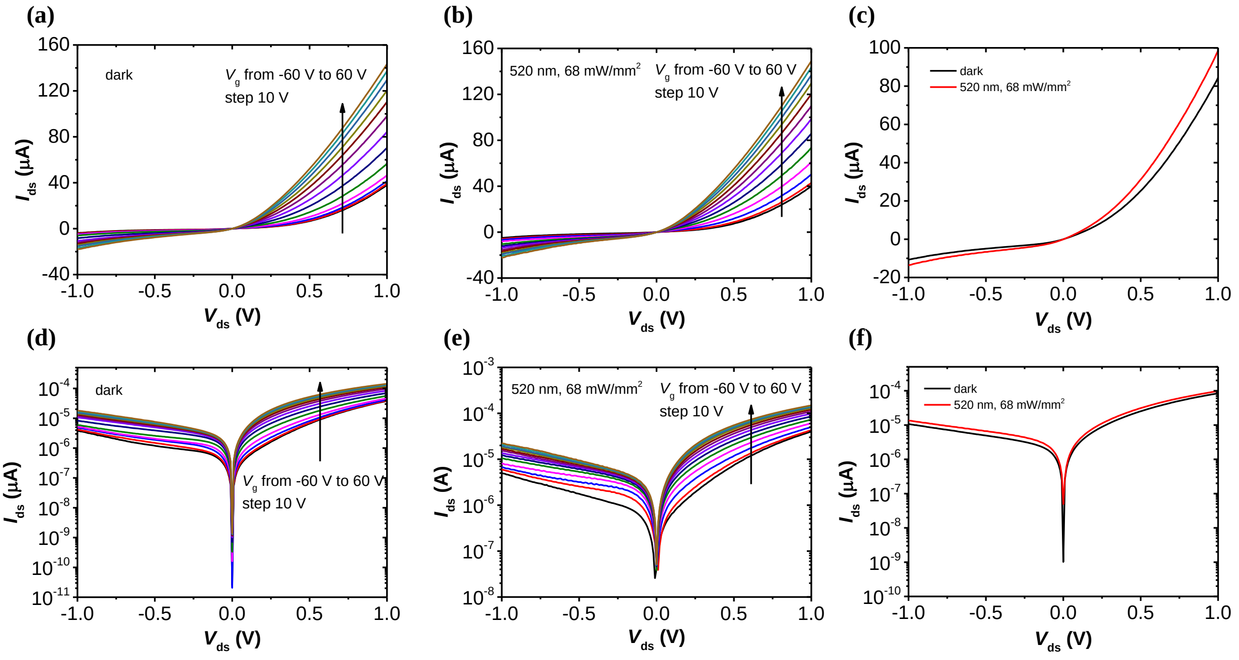

Supplementary Figure 19. Photoresponse of the normal MoS_2/AsP pn heterodiode. I_{ds}-V_{ds} curves of the normal MoS_2/AsP pn heterodiode under dark in (a) linear scale and (d) semi-logarithmic scale. I_{ds}-V_{ds} curves of the normal MoS_2/AsP pn heterodiode under 520 nm laser illumination in (b) linear scale and (e) semi-logarithmic scale. I_{ds}-V_{ds} curves of the normal MoS_2/AsP pn heterodiode at V_g=0 V under dark and 520 nm laser illumination, respectively, in (c) linear scale and (f) semi-logarithmic scale.

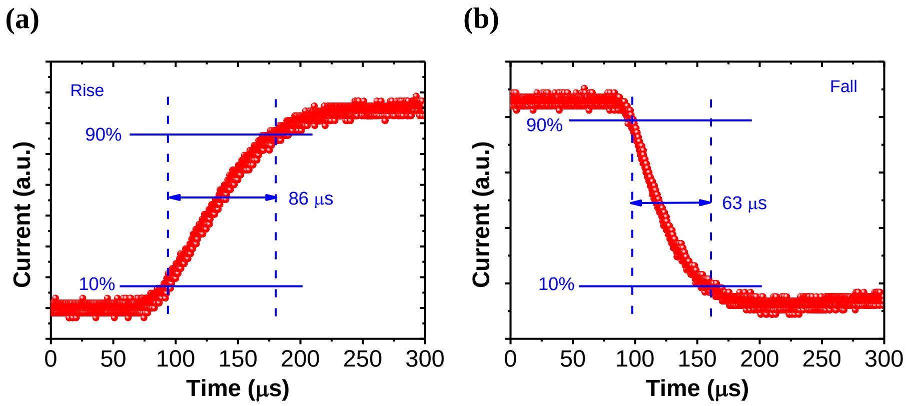

Supplementary Figure 20. Time-resolved photoresponse of the normal MoS_2/AsP pn heterodiode at V_{ds} =0 V. (a) Rise process and (b) fall process, showing the rise time of 86 μs and fall time of 63 μs, respectively.

Effect of thickness on the photovoltaic response of MoS_2/AsP heterodiode

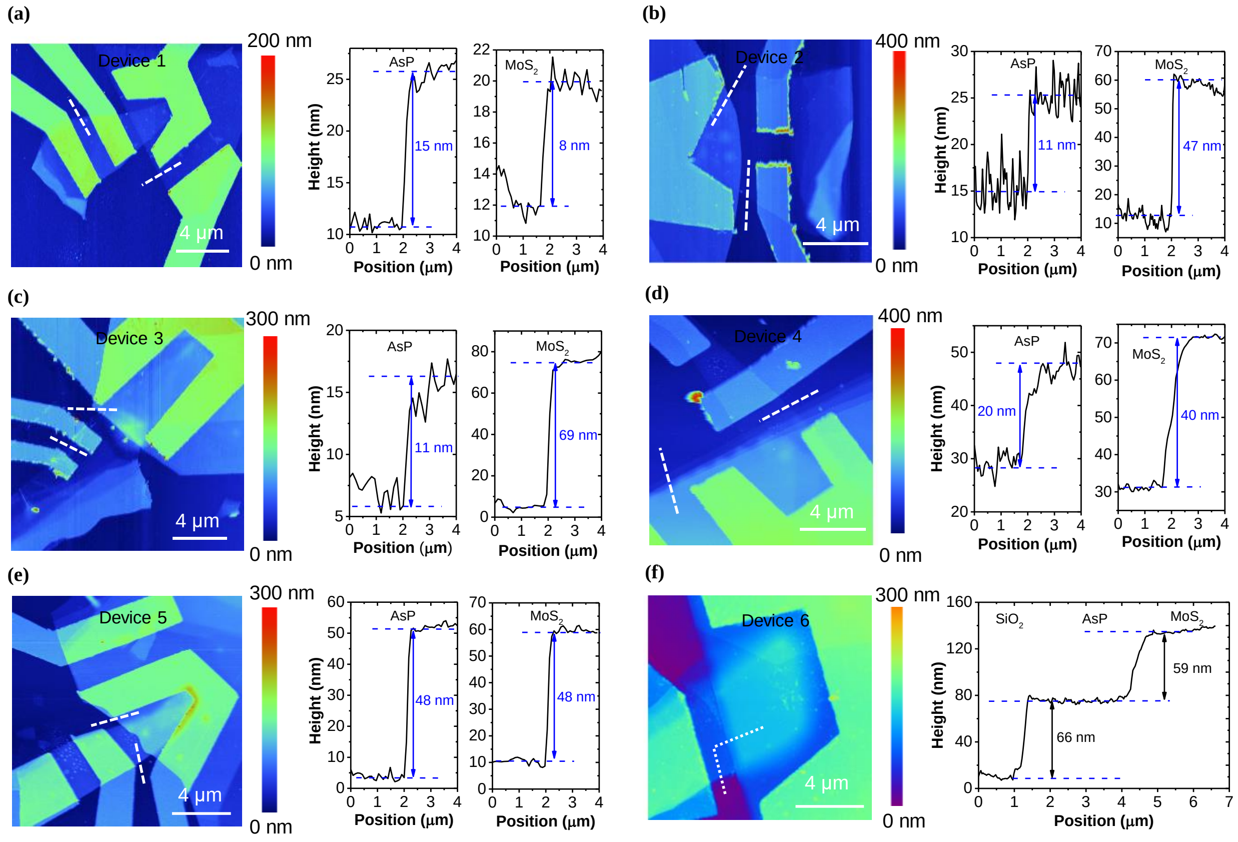

Supplementary Figure 21. AFM images and corresponding thickness profiles of MoS_2 and AsP flakes of all six devices. (a) Device 1. (b) Device 2. (c) Device 3. (d) Device 4. (e) Device 5. (f) Device 6.

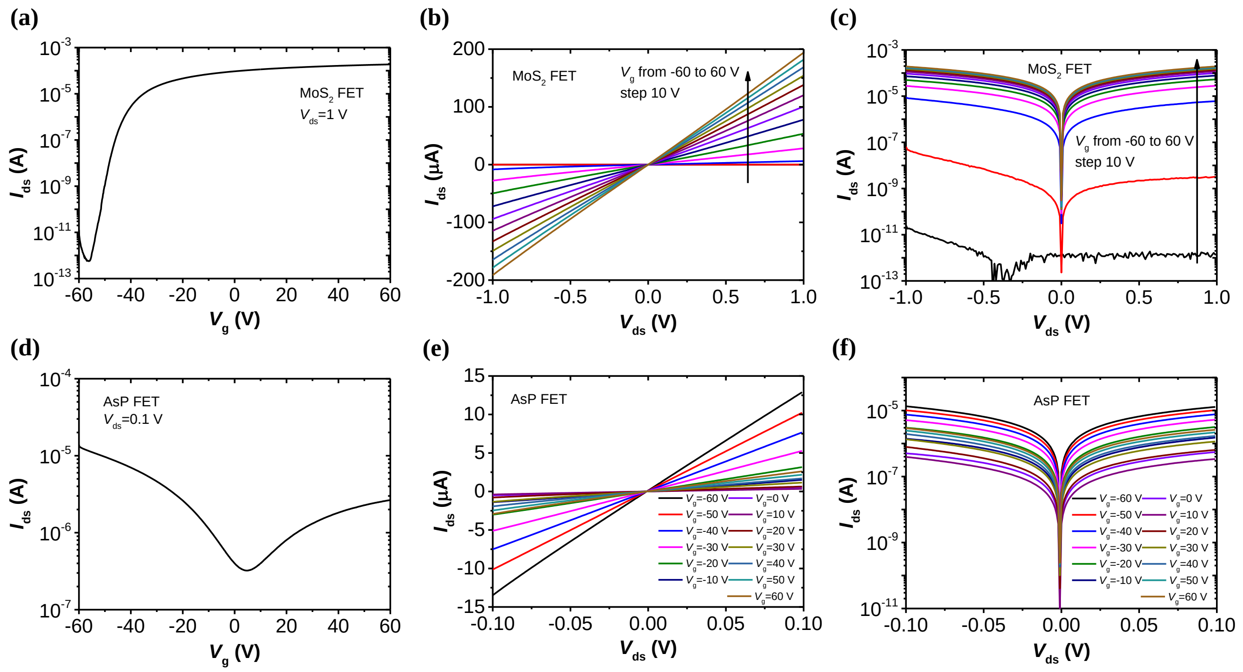

Supplementary Figure 22. Electrical characterizations of individual MoS_2 and AsP FETs from Device 1. (a) Transfer curve of the MoS_2 FET in semi-logarithmic scale. I_{ds}-V_{ds} curves of the MoS_2 FET under different gate voltages in (b) linear scale and (c) semi-logarithmic scale. (d) Transfer curve of the AsP FET in semi-logarithmic scale. I_{ds}-V_{ds} curves of the AsP FET under different gate voltages in (e) linear scale and (f) semi-logarithmic scale.

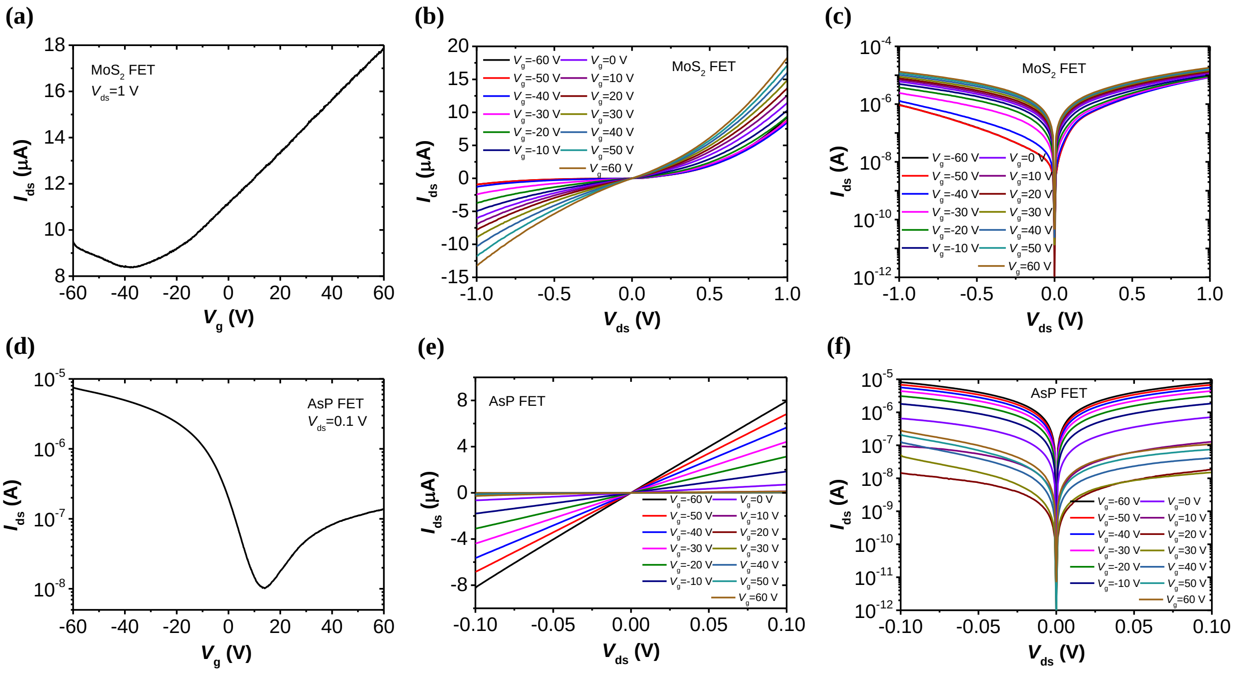

Supplementary Figure 23. Electrical characterizations of individual MoS_2 and AsP FETs from Device 2. (a) Transfer curve of the MoS_2 FET in linear scale. I_{ds}-V_{ds} curves of the MoS_2 FET under different gate voltages in (b) linear scale and (c) semi-logarithmic scale. (d) Transfer curve of the AsP FET in semi-logarithmic scale. I_{ds}-V_{ds} curves of the AsP FET under different gate voltages in (e) linear scale and (f) semi-logarithmic scale.

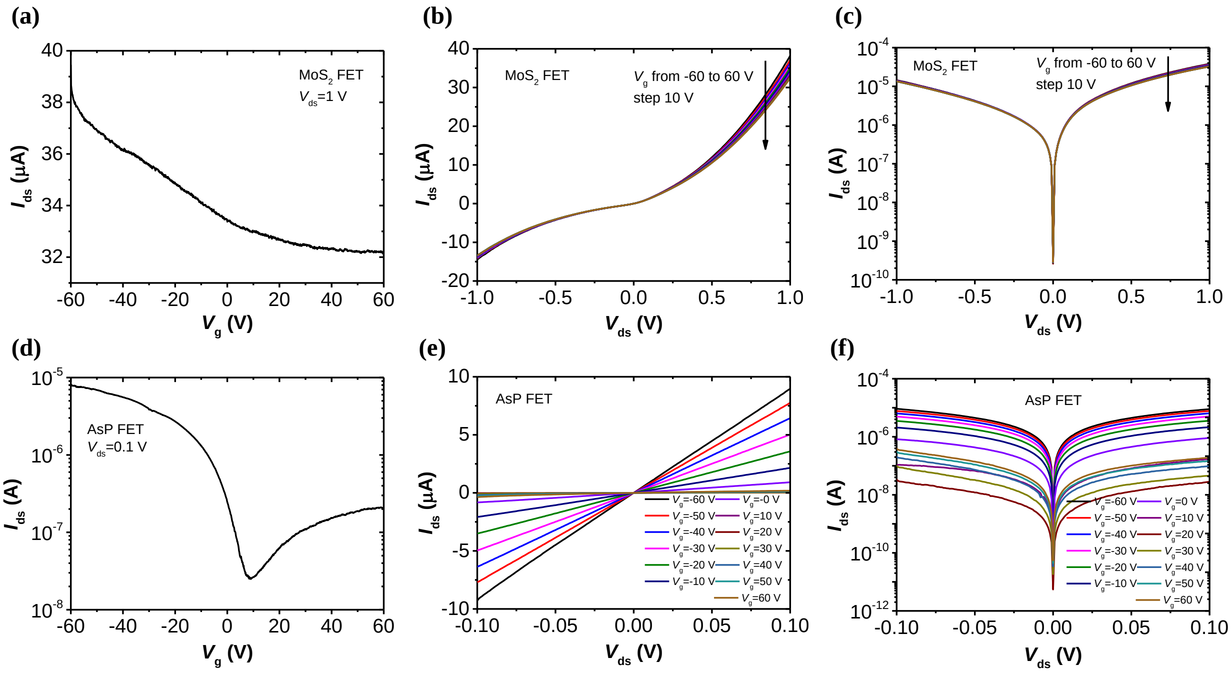

Supplementary Figure 24. Electrical characterizations of individual MoS_2 and AsP FETs from Device 3. (a) Transfer curve of the MoS_2 FET in linear scale. I_{ds}-V_{ds} curves of the MoS_2 FET under different gate voltages in (b) linear scale and (c) semi-logarithmic scale. (d) Transfer curve of the AsP FET in semi-logarithmic scale. I_{ds}-V_{ds} curves of the AsP FET under different gate voltages in (e) linear scale and (f) semi-logarithmic scale.

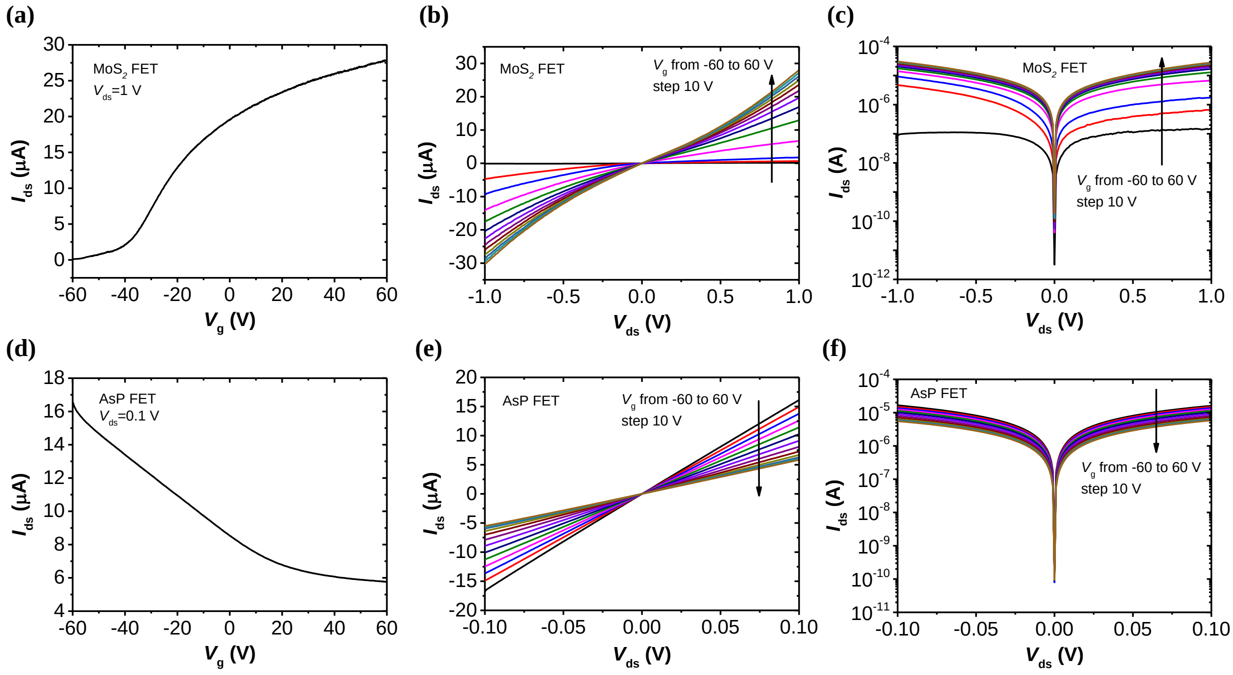

Supplementary Figure 25. Electrical characterizations of individual MoS_2 and AsP FETs from Device 4. (a) Transfer curve of the MoS_2 FET in linear scale. I_{ds}-V_{ds} curves of the MoS_2 FET under different gate voltages in (b) linear scale and (c) semi-logarithmic scale. (d) Transfer curve of the AsP FET in linear scale. I_{ds}-V_{ds} curves of the AsP FET under different gate voltages in (e) linear scale and (f) semi-logarithmic scale.

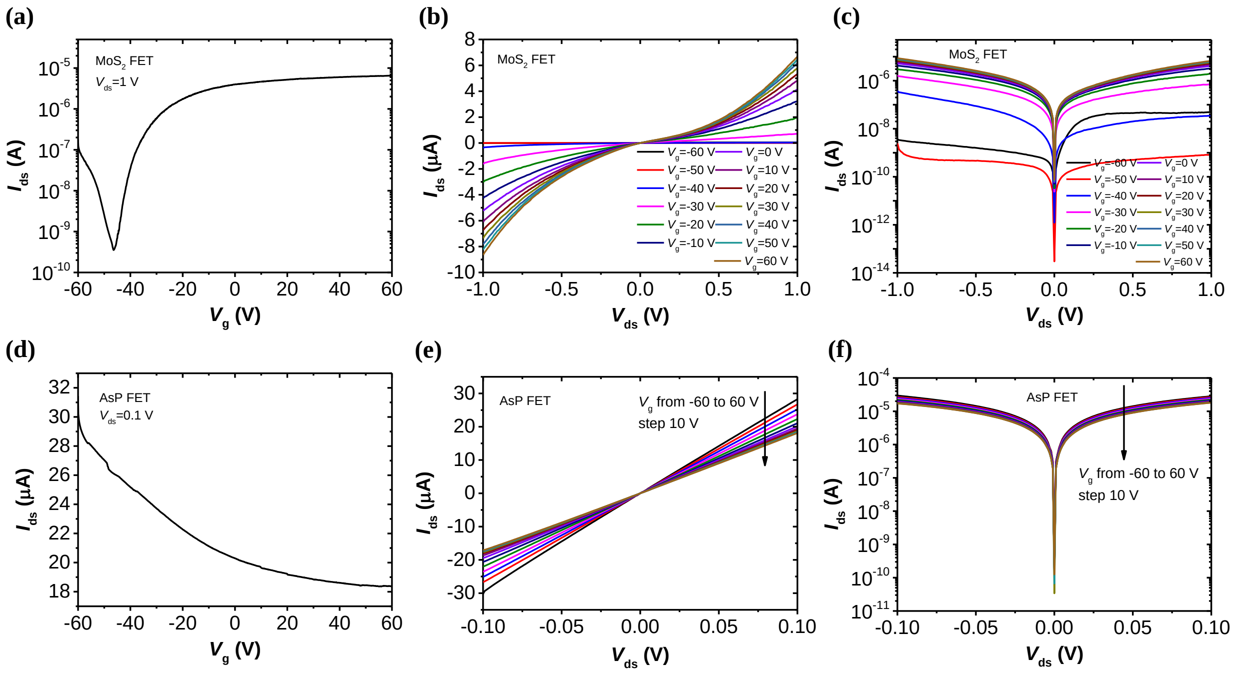

Supplementary Figure 26. Electrical characterizations of individual MoS_2 and AsP FETs from Device 5. (a) Transfer curve of the MoS_2 FET in semi-logarithmic scale. I_{ds}-V_{ds} curves of the MoS_2 FET under different gate voltages in (b) linear scale and (c) semi-logarithmic scale. (d) Transfer curve of the AsP FET in linear scale. I_{ds}-V_{ds} curves of the AsP FET under different gate voltages in (e) linear scale and (f) semi-logarithmic scale.

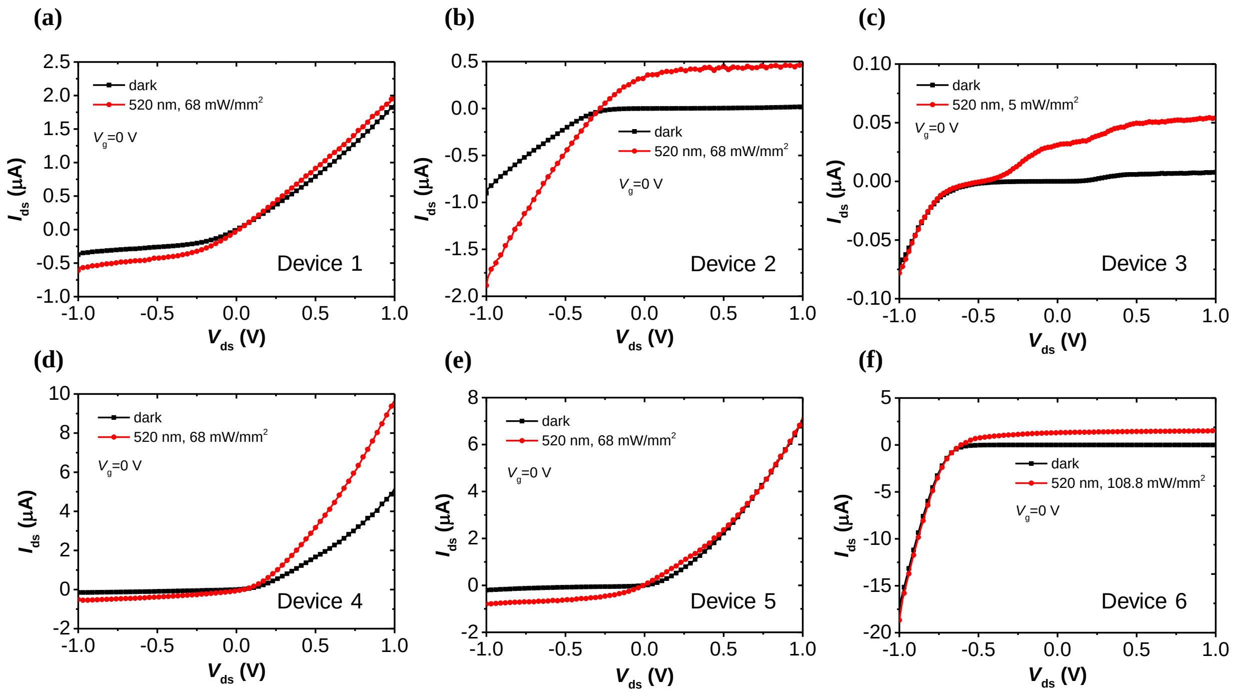

Supplementary Figure 27. I_{ds}-V_{ds} curves of the MoS_2/AsP vdWHs diodes under dark (black line with rectangle) and 520 nm laser illumination (red line with circle), respectively, in linear scale. (a) Device 1. (b) Device 2. (c) Device 3. (d) Device 4. (e) Device 5. (f) Device 6.

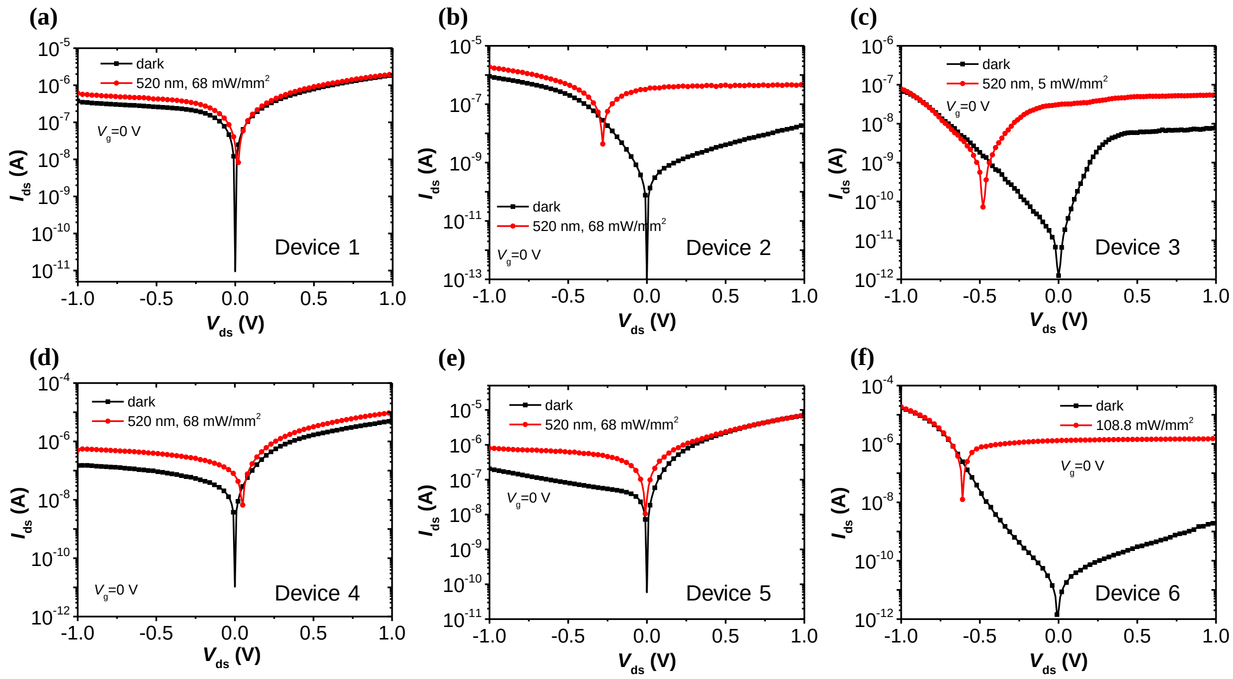

Supplementary Figure 28. I_{ds}-V_{ds} curves of the MoS_2/AsP vdWHs diodes under dark (black line with rectangle) and 520 nm laser illumination (red line with circle), respectively, in semi-logarithmic scale. (a) Device 1. (b) Device 2. (c) Device 3. (d) Device 4. (e) Device 5. (f) Device 6.

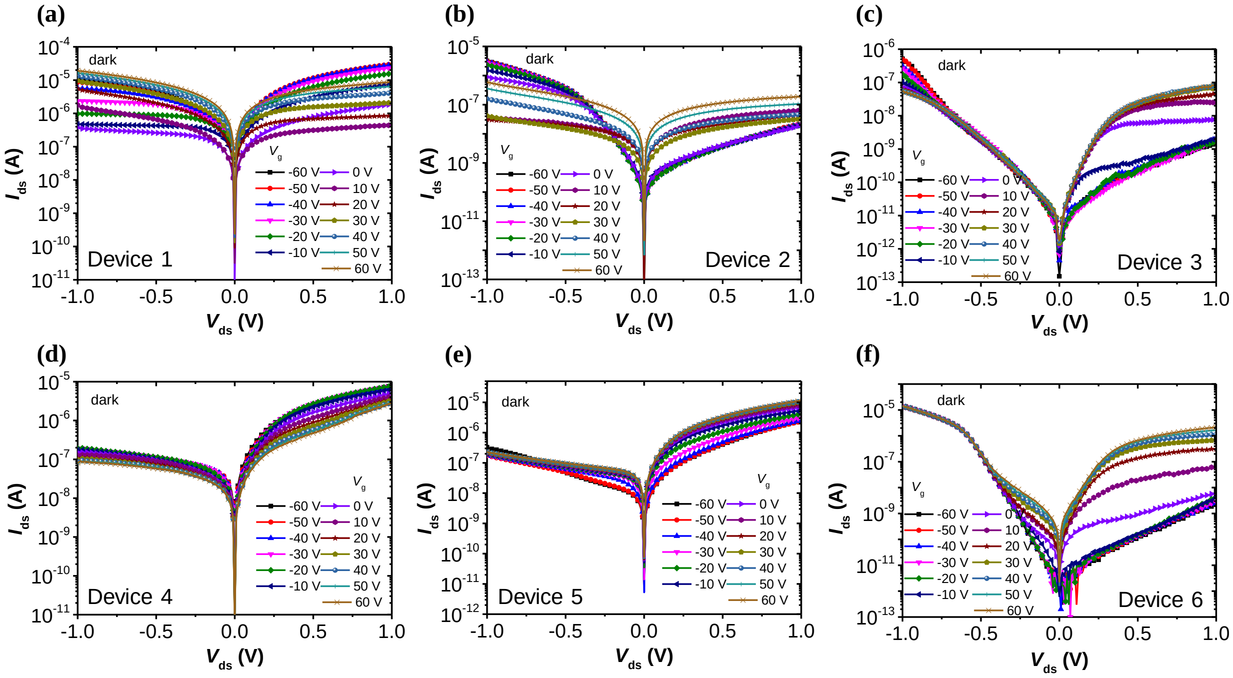

Supplementary Figure 29. I_{ds}-V_{ds} curves of the MoS_2/AsP vdWHs diodes under dark at different gate voltages in semi-logarithmic scale. (a) Device 1. (b) Device 2. (c) Device 3. (d) Device 4. (e) Device 5. (f) Device 6.

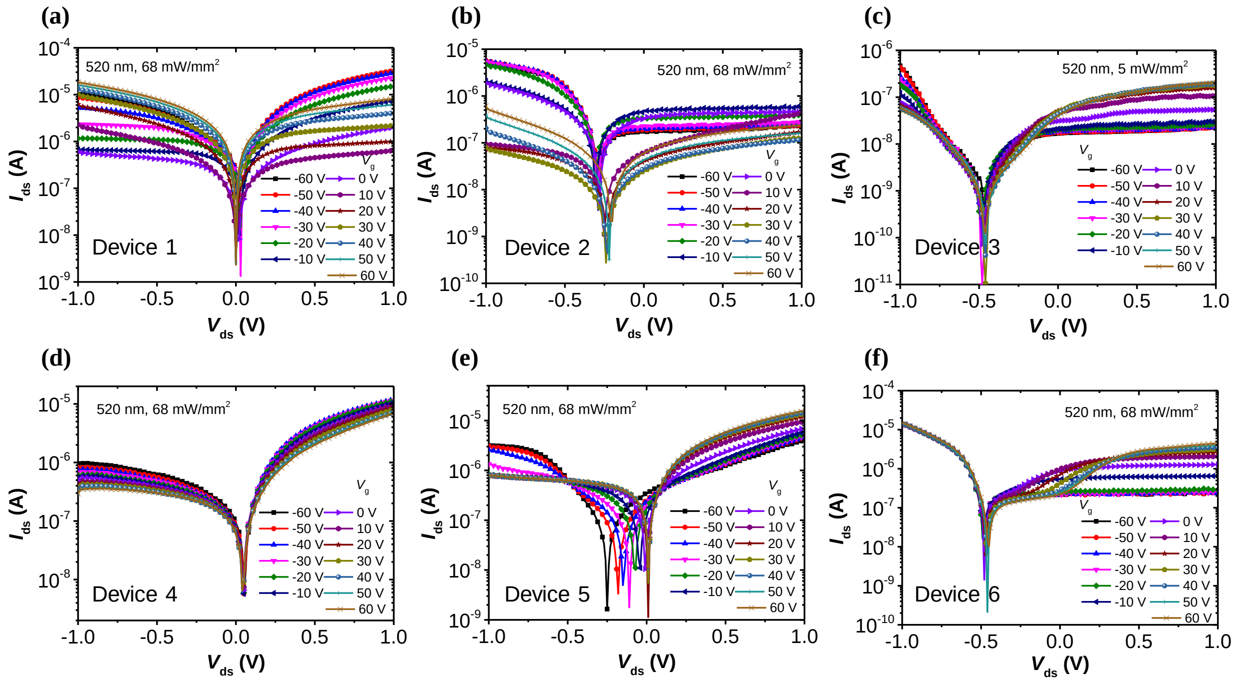

Supplementary Figure 30. I_{ds}-V_{ds} curves of the MoS_2/AsP vdWHs diodes under 520 nm laser illumination at different gate voltages in semi-logarithmic scale. (a) Device 1. (b) Device 2. (c) Device 3. (d) Device 4. (e) Device 5. (f) Device 6.

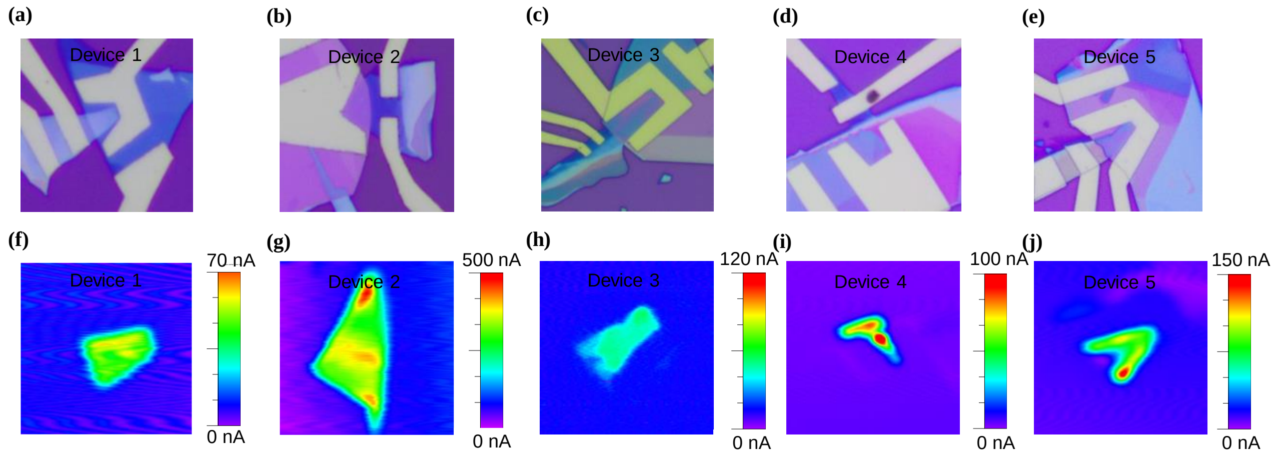

Supplementary Figure 31. Optical images of the front five MoS_2/AsP vdWHs devices. (a) Device 1. (b) Device 2. (c) Device 3. (d) Device 4. (e) Device 5. Scanning photocurrent mappings of the front five MoS_2/AsP vdWHs devices at V_{ds}=0 V under 520 nm laser illumination. (f) Device 1. (g) Device 2. (h) Device 3. (i) Device 4. (j) Device 5. The scanning photocurrent mappings demonstrate that the photovoltaic response of all the devices come from the heterojunction.

MoS_2/BP vdWHs photodiode with unilateral depletion region

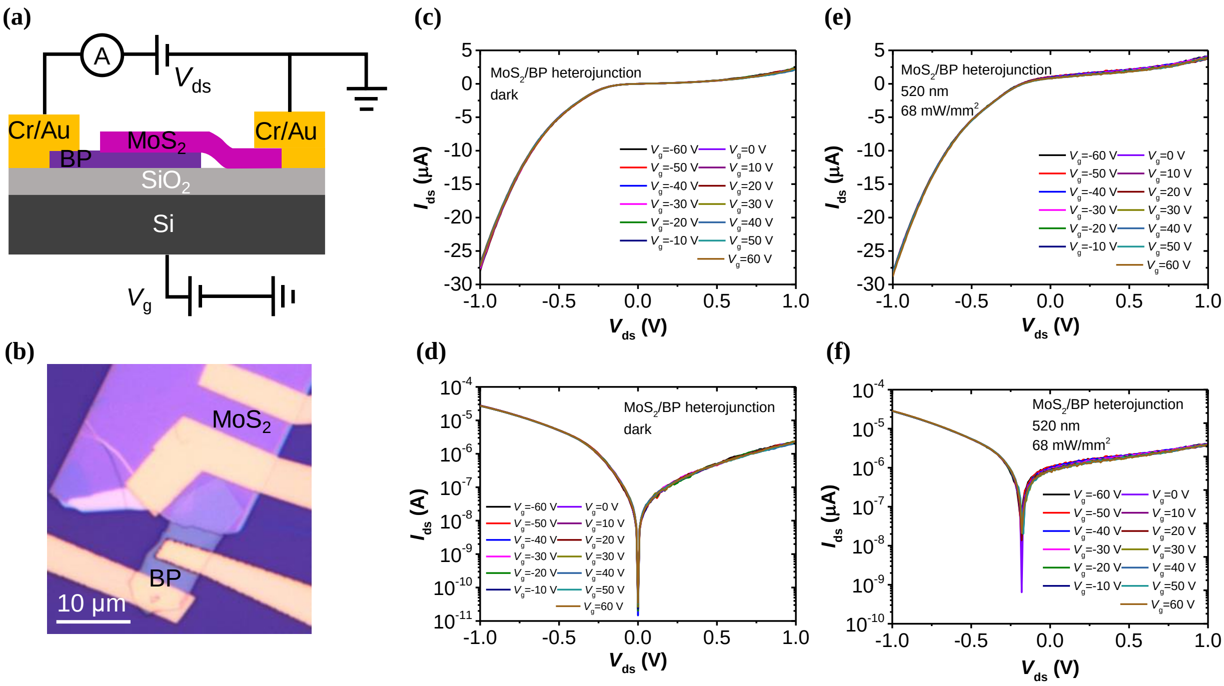

Supplementary Figure 32. Electrical and photoresponse characterizations of MoS_2/BP pp$^+$ heterojunction. (a) Schematic of the fabricated MoS_2/BP pp$^+$ heterojunction device for electrical measurement. (b) Optical image of the fabricated MoS_2/AsP pp$^+$ heterojunction device. I_{ds}-V_{ds} curves of the MoS_2/BP pp$^+$ heterojunction device at different gate voltages varying from -60 to 60 V in (c) linear and (d) semi-logarithmic scale. I_{ds}-V_{ds} curves of the MoS_2/BP pp$^+$ heterojunction device under 520 nm laser illumination at different gate voltages varying from -60 to 60 V in (e) linear and (f) semi-logarithmic scale.

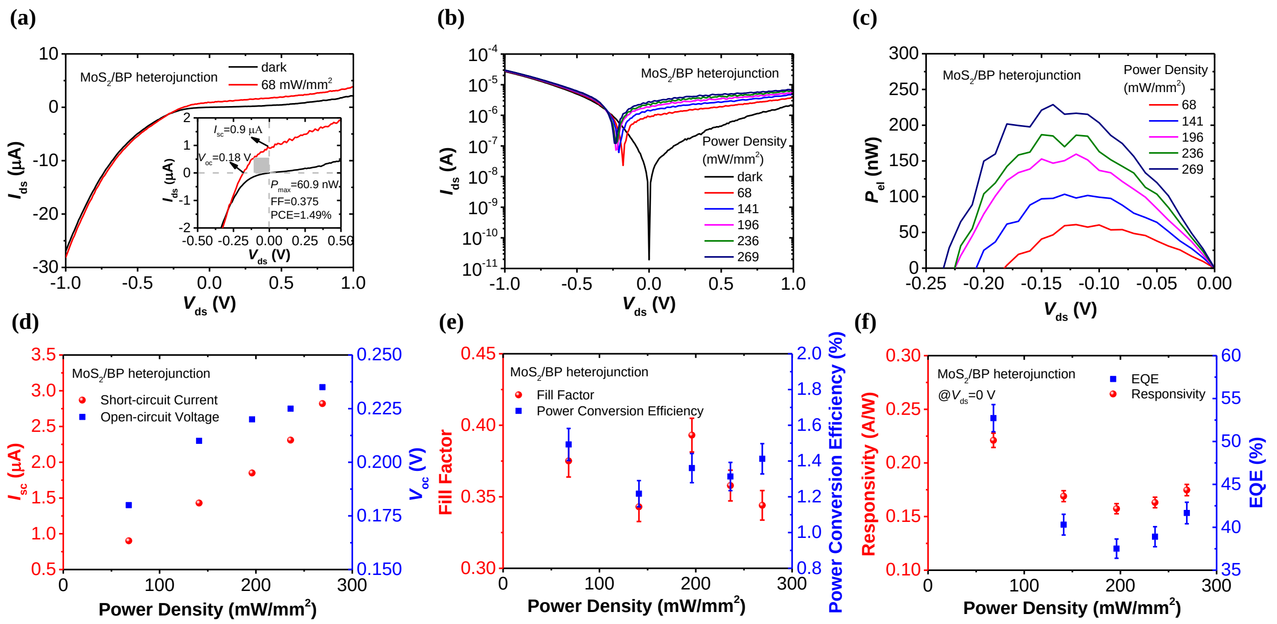

Supplementary Figure 33. Photovoltaic response of the MoS_2/BP vdWHs diode. (a) I_{ds}-V_{ds} curves of the MoS_2/BP vdWHs diode under dark (black line) and 520 nm laser illumination (red line), respectively. The inset is the enlarged I_{ds}-V_{ds} curves showing the photovoltaic response with the performance parameters. (b) I_{ds}-V_{ds} curves of the MoS_2/BP vdWHs diode under 520 nm laser illumination with different power densities. (c) Output electrical power P_{el} as a function of V_{ds}. (d) Power dependent short-circuit current I_{sc} and open-circuit voltage V_{oc}. (e) Power dependent fill factor (FF) and power conversion efficiency (PCE). (f) Power dependent responsivity and external quantum efficiency (EQE). Error bars represent standard deviation.

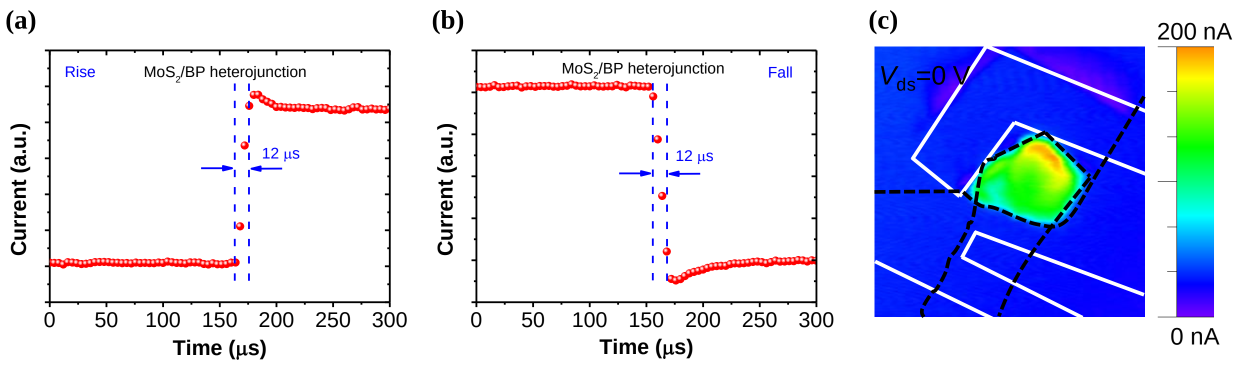

Supplementary Figure 34. Response time measurement and photocurrent mapping of the MoS_2/BP vdWHs diode. Time-resolved photoresponse of the MoS_2/BP vdWHs diode at V_{ds} =0, (a) rise and (b) fall process. (c) Scanning photocurrent mapping of the MoS_2/BP vdWHs diode under 520 nm laser illumination at V_{ds} =0 V.

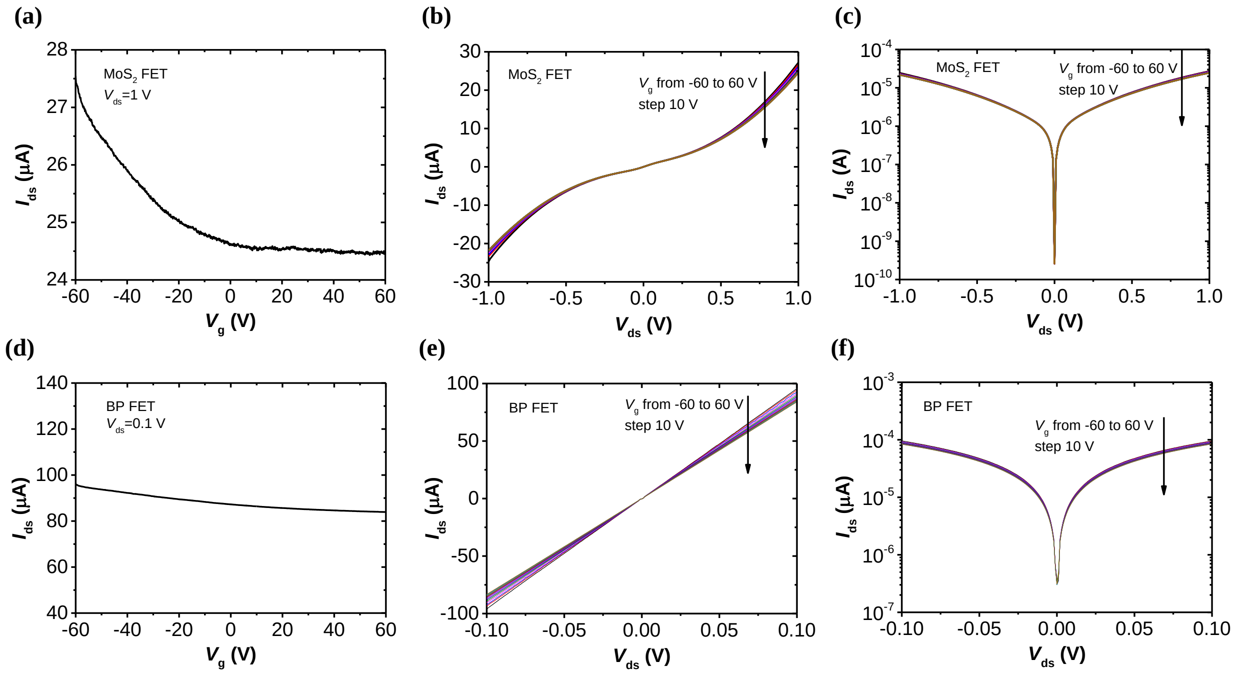

Supplementary Figure 35. Electrical characterizations of individual MoS_2 and BP FETs. (a) Transfer curve of MoS_2 FET. (b) I_{ds}-V_{ds} curve of MoS_2 FET under different gate voltages in linear scale. (c) I_{ds}-V_{ds} curve of MoS_2 FET under different gate voltages in semi-logarithmic scale. (d) Transfer curve of BP FET. (e) I_{ds}-V_{ds} curve of BP FET under different gate voltages in linear scale. (f) I_{ds}-V_{ds} curve of BP FET under different gate voltages in semi-logarithmic scale.

Supplementary Note 1

Definition of unilateral depletion region junction

According to the book,[1] the heterojunctions can be categorized into four types as pn, np, nn and pp junction. Based on the Anderson model, both sides near the interface can be either a carrier depletion region or a carrier accumulation region depending on the relative Fermi levels in the two parts. As the Supplementary Figure 1 shows:

1, if both sides near the interface are carrier-depleted, then we define this heterojunction as bilateral depletion region junction.

2, if one side near the interface is carrier-depleted while the other side is carrier-accumulated, then we define this heterojunction as unilateral depletion region junction.

3, if both sides near the interface are carrier-accumulated, then we define this heterojunction as bilateral accumulation region junction.

Under this classification of heterojunctions, only pp or nn heterojunction can achieve the heterodiode with a unilateral depletion region. pn and np heterojunction belongs to either bilateral depletion region junction or bilateral accumulation region junction.

Supplementary Note 2

The gate dependent photovoltaic response of the MoS_2/AsP vdWHs diode under 520 nm laser illumination with the power density of 68 mW/mm^2 are measured and shown in Supplementary Figure 9. The short-circuit current I_{sc} shows a Gaussian like distribution with a peak at V_g=10 V while the open-circuit voltage V_{oc} decreases slowly with increasing V_g. The Gaussian like distribution of short-circuit current I_{sc} is consistent with previous report of a MoS_2/WSe_2 heterojunction, which is attributed to the Langevin and or Shockley–Read–Hall (SRH) recombination at the heterointerface.[2] The weak gate-dependent V_{oc} is due to the stable Fermi level difference at the heterojunction, which is reflected by the weak gate control of both the thick AsP flake and the thick MoS_2 flake.

Supplementary Note 3

According to the reference,[3] the open-circuit voltage V_{oc} can be expressed:

$$V_{oc} = \frac{n_{oc}k_bT}{q}\ln(\frac{I_{sc}}{I_o} + 1) \quad (1)$$

Where n_{oc} is the ideality factor, k_b is the Boltzmann constant, T is the absolute temperature, q is the elemental charge. Since I_{sc} is almost linearly proportional to P_{in} (Figure 5(d)), equation 1 can be modified as follows:

$$V_{oc} = \frac{n_{oc}k_bT}{q}\ln(\frac{P_{in}}{P_o} + 1) \quad (2)$$

Where P_{in} is the incident power. This equation indicates that the open-circuit voltage increases with the incident power and has a logarithmic relationship with incident power. Our experimental data fits very well by equation 2, as shown in Supplementary Figure 14.

Supplementary Note 4

To verify the p-type conduction of the thick MoS_2 flake is not an accidental phenomenon, we have fabricated many MoS_2 FETs with different thicknesses and measured the transfer curves independently. As presented in Supplementary Figure 15, the transfer curves show that when the thickness is large (in the range of 50-80 nm), the MoS_2 flakes indeed show p-type conductions. The p-type conduction of thick MoS_2 flakes has been reported in literatures by other groups and may be attributed to the impurities-induced doping effect or the defect-induced doping effect (e.g. S adatoms).[4,5] When the thickness decreases, the conduction type of MoS_2 flakes changes from p-type to bipolar and finally n-type. This transition is likely caused by the competition between the impurities-induced p-doping effect in bulk and the substrate-induced S vacancy n-doping effect in thin flakes.[4]

Supplementary Note 5

Effect of thickness on the photovoltaic response of MoS_2/AsP heterodiode

Since the conduction type of both MoS_2 and AsP are thickness-dependent, especially MoS_2 flake, the thickness of MoS_2 should have a significant effect on the photovoltaic performance of the MoS_2/AsP heterodiode. Based on this knowledge, we have fabricated six MoS_2/AsP heterojunction devices to investigate the effect of thickness on the device performance, as classified in the below Supplementary Table 1. The thicknesses of MoS_2 and AsP flakes are obtained by AFM measurements. The conduction type of MoS_2 and AsP flakes are confirmed by transfer curves of individual FET devices.

The photovoltaic response of the six devices at zero gate voltage are shown in Supplementary Fig. 27 and 28. It is obvious that Device 3 and 6 (pp^+ heterojunctions with unilateral depletion regions) exhibit the most efficient photovoltaic response with the largest open-circuit voltages and short-circuit currents (normalized by incident power). Device 2 and 5 exhibit reduced/intermediate photovoltaic response while Device 1 and 4 (normal pn heterojunctions) exhibit the weakest photovoltaic response.

The photovoltaic response of the devices can be modulated by gate voltage, especially for the device with bipolar MoS_2. The detailed gate modulated photovoltaic response of each device are shown in Supplementary Fig. 29 and 30. The open-circuit voltage and short-circuit current are modulated significantly by gate voltage in Device 2 and 5 (heterojunctions with bipolar MoS_2) while this gate modulation effect in other four devices are weak.

Based on these results, conclusions can be obtained as follows:

1, conduction type of MoS_2 flake is highly thickness-dependent, and thickness has a significant effect on the photovoltaic response. Thick MoS_2 flake with p-type conduction is good for photovoltaics.

2, conduction type of AsP flake is highly thickness-dependent, but the thickness has a weak effect on the photovoltaic response.

We are aware that absorption efficiency is thickness-dependent, thus the quantum efficiency and power conversion efficiency are thickness-related. However, comparing Device 1, 2 and 3, or 4, 5 and 6, we can find that the thickness of MoS_2 is changed by less than one order of magnitude, but the photovoltaic efficiency is changed by more than one order of magnitude as seen in Supplementary Table 2. We can conclude that the thickness-related absorption efficiency of MoS_2 is not the major

reason for the large performance variation. Instead, the thickness-dependent band profile of the heterojunction is the key factor determining the photovoltaic efficiency of the MoS_2/AsP heterodiode.

Supplementary Note 6

MoS_2/BP vdWHs photodiode with unilateral depletion region

The vdWHs photodiodes with unilateral depletion region band structure can be applied to other 2D semiconductors, for example BP (similar to AsP). A MoS_2/BP vdWHs photodiode with unilateral depletion region was successfully fabricated and demonstrated similar photovoltaic response as shown in Supplementary Figure 32-35. The *I-V* curve shows a backward diode like characteristic, similar to that of MoS_2/AsP heterojunction in the main manuscript. This MoS_2/BP pp^+ heterojunction with unilateral depletion region also shows an efficient photovoltaic response with an open-circuit voltage of 0.24 V and an external quantum efficiency of 53%. The response is fast with both rise and fall time of 12 μs. In comparison with MoS_2/AsP vdWHs photodiode, the MoS_2/BP vdWHs photodiode exhibits lower photovoltaic efficiency and device performance, which may be attributed to a different band alignment of the heterojunction and/or the unoptimized band profile of the device.

Supplementary Table 1. Classifications of MoS_2/AsP heterojunctions

	n-type MoS_2 (<40 nm)	bipolar MoS_2 (~45 nm)	p-type MoS_2 (>55 nm)
Bipolar AsP (<15nm)	np or nn heterojunction (Device 1)	pp, pn, np, or nn heterojunction (Device 2)	pp^+ or pn heterojunction (Device 3)
p-type AsP (>20 nm)	pn heterojunction (Device 4)	np^+ or pp^+ heterojunction (Device 5)	pp^+ heterojunction (Device 6, main device in manuscript)

Supplementary Table 2. External quantum efficiency (EQE) and power conversion efficiency (PCE) of six MoS_2/AsP heterojunction devices

	Device 1 (V_g=0 V)	Device 2 (V_g=0 V)	Device 3 (V_g=0 V)	Device 4 (V_g=0 V)	Device 5 (V_g=0 V)	Device 5 (V_g=-60 V)	Device 6 (V_g=0 V)
thickness (nm)	8/15	47/11	69/11	40/20	48/48	48/48	59/66
EQE	9.4%	54.4%	42%	26%	4.6%	56.3 %	71%
PCE	0.014%	2.1%	2.2%	0.14%	NA	1.5%	9%

Supplementary Table 3. Comparison of the figure of merits for photovoltaic detectors or solar cells based on 2D vdWHs.

Device structure	Measurement condition	V_{oc} (V)	I_{sc}	Response time	EQE (%)	FF	PCE (%)	Ref.
MoS_2/WSe_2	V_g=0 V, 532 nm laser	0.5	70 nA	/	34	/	/	[2]
MoS_2/WSe_2	V_g=0 V, 514 nm laser	0.27	200 nA	<100 µs	12	/	0.4	[6]
MoS_2/WSe_2	V_g=-50 V, halogen lamp	0.53	50 pA	/	1.5	0.5	0.2	[3]
MoS_2/WSe_2	V_g=0 V, 633 nm laser	0.38	20 µA	/	42	0.42	3.4	[7]
BP/MoS_2	V_g=-40 V, 633 nm laser	0.3	20 nA	/	0.3	0.5	0.57	[8]
$MoTe_2/MoS_2$	V_g=0 V, 800 nm laser	0.3	200 nA	25 ms	6	/	/	[9]
$GaTe/MoS_2$	V_g=70 V, 437 nm laser	0.224	4.5 nA	7 ms	61.7	0.25	0.42	[10]
GaTe/InSe	V_g=0 V, 405 nm laser	0.25	28 pA	20 µs	4.2	0.25	0.1	[11]
BP/WSe_2	V_g=-10 V, 630 nm laser	0.35	4.6 nA	<0.5 s	23	0.41	1.7	[12]
BP/WS_2	V_g=-40 V, AM 1.5 light	0.19	21 nA	3 ms	/	0.33	4.6	[13]
**MoS_2/AsP**	**V_g=0 V, 520 nm laser**	**0.61**	**1.3 µA**	**9 µs**	**71**	**0.5**	**9**	**This work**

Supplementary References

1 Sharma, B. L. & Purohit, R. K. *Semiconductor Heterojunctions*. Vol. 5 (Elsevier, 2015).

2 Lee, C.-H. *et al.* Atomically thin p–n junctions with van der Waals heterointerfaces. *Nature Nanotechnology* **9**, 676 (2014).

3 Furchi, M. M., Pospischil, A., Libisch, F., Burgdörfer, J. & Mueller, T. Photovoltaic Effect in an Electrically Tunable van der Waals Heterojunction. *Nano Letters* **14**, 4785-4791 (2014).

4 Liu, Y. *et al.* Vertical Charge Transport and Negative Transconductance in Multilayer Molybdenum Disulfides. *Nano Letters* **17**, 5495-5501 (2017).

5 Kc, S., Longo, R. C., Addou, R., Wallace, R. M. & Cho, K. Impact of intrinsic atomic defects on the electronic structure of MoS_2 monolayers. *Nanotechnology* **25**, 375703 (2014).

6 Cheng, R. *et al.* Electroluminescence and Photocurrent Generation from Atomically Sharp WSe_2/MoS_2 Heterojunction p–n Diodes. *Nano Letters* **14**, 5590-5597 (2014).

7 Wong, J. *et al.* High Photovoltaic Quantum Efficiency in Ultrathin van der Waals Heterostructures. *ACS Nano* **11**, 7230-7240 (2017).

8 Deng, Y. *et al.* Black Phosphorus–Monolayer MoS_2 van der Waals Heterojunction p–n Diode. *ACS Nano* **8**, 8292-8299 (2014).

9 Pezeshki, A., Shokouh, S. H. H., Nazari, T., Oh, K. & Im, S. Electric and Photovoltaic Behavior of a Few-Layer α-$MoTe_2/MoS_2$ Dichalcogenide Heterojunction. *Advanced Materials* **28**, 3216-3222 (2016).

10 Wang, F. *et al.* Tunable GaTe-MoS_2 van der Waals p–n Junctions with Novel Optoelectronic Performance. *Nano Letters* **15**, 7558-7566 (2015).

11 Feng, W. *et al.* A fast and zero-biased photodetector based on GaTe–InSe vertical 2D p–n heterojunction. *2D Materials* **5**, 025008 (2018).

12 Li, D., Wang, B., Chen, M., Zhou, J. & Zhang, Z. Gate-Controlled BP–WSe_2 Heterojunction Diode for Logic Rectifiers and Logic Optoelectronics. *Small* **13**, 1603726 (2017).

13 Kwak, D.-H., Ra, H.-S., Jeong, M.-H., Lee, A. Y. & Lee, J.-S. High-Performance Photovoltaic Effect with Electrically Balanced Charge Carriers in Black Phosphorus and WS_2 Heterojunction. *Advanced Materials Interfaces* **5**, 1800671 (2018).
